# Capturing the Alternative Cleavage and Polyadenylation Sites of 14 NAC Genes in *Populus* Using a Combination of 3′-RACE and High-Throughput Sequencing

**DOI:** 10.3390/molecules23030608

**Published:** 2018-03-08

**Authors:** Haoran Wang, Mingxiu Wang, Qiang Cheng

**Affiliations:** The Southern Modern Forestry Collaborative Innovation Center, Nanjing Forestry University, Nanjing 210037, China; njlydxwhr@163.com

**Keywords:** 3′-RACE, high-throughput sequencing, alternative splicing, alternative polyadenylation, *Populus*

## Abstract

Detection of complex splice sites (SSs) and polyadenylation sites (PASs) of eukaryotic genes is essential for the elucidation of gene regulatory mechanisms. Transcriptome-wide studies using high-throughput sequencing (HTS) have revealed prevalent alternative splicing (AS) and alternative polyadenylation (APA) in plants. However, small-scale and high-depth HTS aimed at detecting genes or gene families are very few and limited. We explored a convenient and flexible method for profiling SSs and PASs, which combines rapid amplification of 3′-cDNA ends (3′-RACE) and HTS. Fourteen NAC (NAM, ATAF1/2, CUC2) transcription factor genes of *Populus trichocarpa* were analyzed by 3′-RACE-seq. Based on experimental reproducibility, boundary sequence analysis and reverse transcription PCR (RT-PCR) verification, only canonical SSs were considered to be authentic. Based on stringent criteria, candidate PASs without any internal priming features were chosen as authentic PASs and assumed to be PAS-rich markers. Thirty-four novel canonical SSs, six intronic/internal exons and thirty 3′-UTR PAS-rich markers were revealed by 3′-RACE-seq. Using 3′-RACE and real-time PCR, we confirmed that three APA transcripts ending in/around PAS-rich markers were differentially regulated in response to plant hormones. Our results indicate that 3′-RACE-seq is a robust and cost-effective method to discover SSs and label active regions subjected to APA for genes or gene families. The method is suitable for small-scale AS and APA research in the initial stage.

## 1. Introduction

For eukaryotes, precise splicing and polyadenylation are required for production of translatable mRNA. In many cases, there is more than one way to choose splice sites (SSs) and polyadenylation sites (PASs), resulting in alternative splicing (AS) and alternative polyadenylation (APA) [1]. Both AS and APA contribute to gene expression regulation and can increase proteome complexity. Accumulating evidence shows that AS and APA play key roles in various biological processes. Thus, dissecting AS and APA events in individual genes and at the transcriptome level have been important approaches in understanding gene functions and regulatory mechanisms [2,3,4,5].

With the widespread application of high-throughput sequencing (HTS) technology, it was shown that AS is prevalent in plants [6,7,8,9,10]. The frequency of detected AS continually increases along with sequencing depth, sampling type diversification and technical advances. For example, an early HTS study of *Arabidopsis* revealed an AS frequency of 42% in multi-exonic genes [6]. Yet, with a normalized library and longer reads, AS frequency in *Arabidopsis* was estimated to be 61% [8]. More recently, analysis on the *Arabidopsis* infected by *Pseudomonas syringae*, for which the sequencing coverage was approximately five-fold over the previous two studies, revealed that even more expressed genes might undergo AS [7]. In maize, based on six billion read pairs derived from 94 libraries of different tissues at different developmental stages, AS events were uncovered in 42% of multi-exonic genes [9]. A more recent study, through deep-sequencing and analysis of 1.3 billion read pairs on drought-stressed maize libraries, identified 48,000 novel alternative isoforms [10].

Transcriptome-wide APA studies, mainly in *Arabidopsis*, have also benefited from HTS technology. Using Poly(A) tag sequencing (PAT-seq), for which RNA was reverse transcribed by anchored oligo(dT), followed by adaptor-mediated second-strand synthesis, PCR amplification and HTS, 70% of *Arabidopsis* genes were identified to have more than one polyadenylation site cluster (PAC), with 17% of the PACs being located in annotated coding sequences (CDSs), introns, or 5′-UTRs [11]. However, although the PAT-seq data were filtered by eliminating PASs with stretches of six or more adenosines, these identified PASs were possibly affected by internal priming. Internal priming is internal poly(A) priming by which oligo(dT) primer generates a high frequency of truncated cDNAs in the process of reverse transcription (RT) [12]. Another study that directly sequenced *Arabidopsis* native RNA revealed that few PASs (1.16%) were located in exons, introns and 5′-UTRs, and such sites revealed by previous PAT-seq workflow were poorly supported by direct RNA sequencing [13]. In addition, direct RNA sequencing also revealed extreme heterogeneity at the 3′-ends of plant mRNAs that differed markedly from those of human mRNAs [13]. This was consistent with previous findings, that plant mRNAs had unique high heterogeneous 3′-ends [14,15].

The aforementioned transcriptome-wide AS and APA studies provide a reference for research into genes of interest. However, small-scale HTS methods aimed at genes and gene families are still necessary. This is because (1) genes of interest may be poorly covered; (2) tissues, developmental stages or treatment conditions may not be included in transcriptome-wide studies; and (3) so far, there is no detailed AS and APA information for most plants.

The RT-PCR sequencing (RT-PCR-seq) method developed by the Encode Project can be flexibly applied to discover AS events [16,17]. However, RT-PCR-seq also has its limitations. In RT-PCR-seq, small regions adjacent to known or putative SSs are enriched by RT-PCR, which is followed by HTS. This approach can discover novel exons in known/putative introns and some alternative donor/acceptor sites in target regions. However, for AS events without any clues, such as the alternative SSs which are not annotated, uncovered by existing expressed sequence tags or absent in other species, RT-PCR-seq is less effective [16].

In this study, we explored an approach that combined traditional rapid amplification of 3′-cDNA ends (3′-RACE) and HTS to simultaneously identify SSs and PASs. The effectiveness of this approach was verified with 14 transcription factor genes in poplar (*Populus trichocarpa*). To avoid false-positive results, filtering criteria were systematically evaluated for identifying authentic SSs and PASs. Although overly stringent filtering criteria may result in some SSs and PASs being overlooked, the increased local sequencing depth of 3′-RACE-seq revealed many novel SSs and PASs, shedding light on regions under active post-transcriptional regulation.

## 2. Results

### 2.1. Target Transcripts Were Effectively Enriched by 3′-RACE

**Figure 1:**
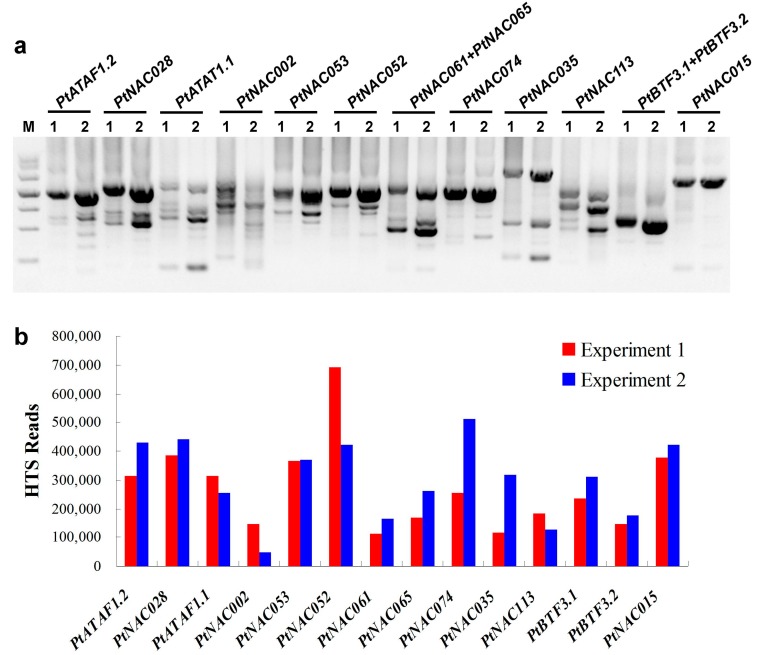
Target transcripts enriched by 3′-RACE. (**a**) Agarose gel electrophoresis of 12 3′-RACEs for 14 poplar NAC transcription factor genes. M, DNA ladder; 1, Experiment 1; 2, Experiment 2; names of target genes are shown at the top of lanes; (**b**) The number of reads mapped to each of 14 target genes.

In traditional 3′-RACE, PCR bias was expected because of the use of two universal adaptor primers and the necessity for two rounds of PCR. In addition, there was no standard for the quantity of template cDNA and the number of cycles for each round of PCR. Thus, two experiments (Experiment 1 and 2) of 3′-RACE were performed. In order to cover most of the target genes, the gene-specific primers were designed adjacent to the initiation codon. A comparable quantity of the twelve 3′-RACE products were obtained from each of Experiment 1 and Experiment 2 (Figure 1a). A total of 24 PCR products represented 3′-RACEs of 14 NAC transcription factor genes of *P. trichocarpa*. These PCR products from Experiment 1 and Experiment 2 were pooled and purified separately, then used for Illumina HTS. The majority of the sequencing reads (70.9% of Experiment 1 and 72.2% of Experiment 2) were mapped to the genome of *P. trichocarpa* (Table S1), then, more than half of these mapped sequences were located in target genes. The average coverage was ~400 reads per base pair of target genes (Figure 1b), indicating significant enrichment of target transcripts by 3′-RACE.

### 2.2. Monte Carlo Effect of Poorly Covered Splice Sites

Deep sequencing revealed 101 SSs for target genes. Thirty-two well-covered SSs, each of which was supported by more than 3800 reads, comprised 3.29–46.6% reads covering its target gene, and all well-covered SSs were canonical GT-AG SSs. Sixty-nine poorly covered SSs, which were supported by 10–1629 reads, comprised 0.001–0.6% reads covering the target gene. There were 40 canonical and 29 non-canonical SSs in these poorly covered SSs.

**Figure 2:**
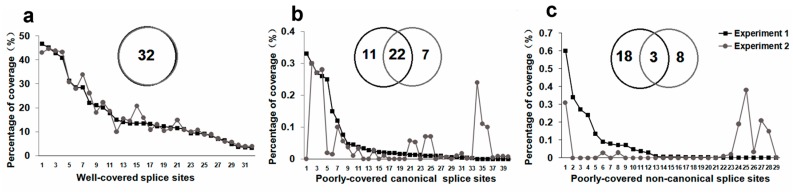
Reproducibility of splice sites (SSs) detected in two 3′-RACE experiments. (**a**) Reproducibility of 32 annotated SSs; (**b**) reproducibility of 40 poorly covered canonical SSs; (**c**) reproducibility of 29 poorly-covered non-canonical SSs. *Y* axis of line chart is the percentage of coverage (reads spanned intron/reads covered target gene × 100 (%)). SSs were arranged from largest to smallest according to the percentage of coverage in Experiment 1. Left circle, SSs detected in Experiment 1; right circle, SSs detected in Experiment 2; the intersection of the left and right circles denotes SSs detected in both experiments.

Although the quantity of starting template and the number of PCR cycles differed, for the well-covered SSs the percentage coverage of each SS was roughly consistent in both experiments (Figure 2a), indicating the linear accumulation of 3′-RACE products. This might be due to the fact that the rate-limiting step of 3′-RACE was linear accumulation of mega-primers complementary to the 3′-end of the target transcripts. In contrast, the poorly covered SSs were often detected in one experiment and absent in another, suggesting a Monte Carlo effect on the PCR (Figure 2b,c). The Monte Carlo effect is that the low-abundance templates are amplified early or late, depending on random factors, which can result in non-reproducibility of amplification [18]. In spite of analogous low coverage of poorly covered canonical and non-canonical SSs, the extent of reproducibility was clear. More than half (22/40) of poorly covered canonical SSs were detected in both experiments (Figure 2b), while only about 1/10 (3/29) of poorly covered non-canonical SSs were present in both experiments (Figure 2c), indicating the presence of more random factors for detecting non-canonical SSs.

### 2.3. Non-Canonical SSs Probably Resulted from PCR Artifacts

**Figure 3:**
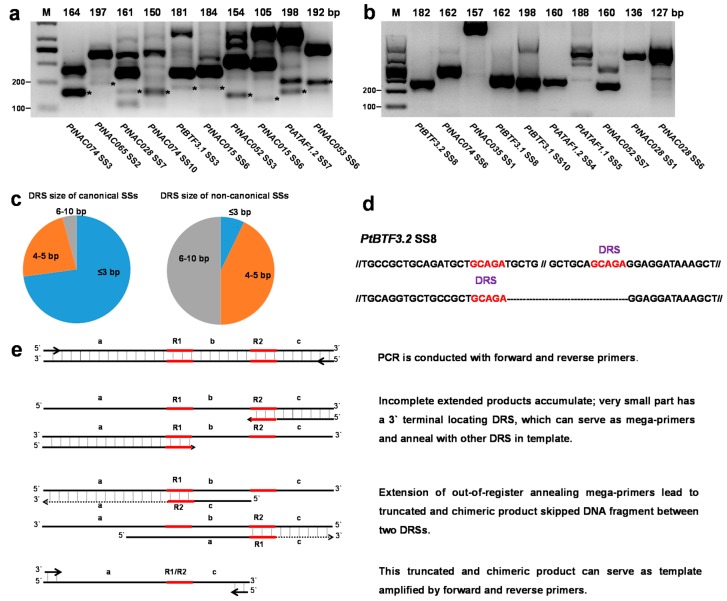
Canonical and non-canonical SSs verified by RT-PCR and the model explaining DRS-mega-primer-mediated amplification. (**a**) Ten canonical SSs verified by RT-PCR. Lanes 2–11 are *PtNAC074* SS3, *PtNAC065* SS2, *PtNAC028* SS7, *PtNAC074* SS10, *PtBTF3.1* SS3, *PtNAC015* SS6, *PtNAC052* SS3, *PtNAC015* SS6, *PtATAF1.2* SS7 and *PtNAC053* SS6; (**b**) Ten non-canonical SSs verified by RT-PCR. Lanes 2–11 are *PtBTF3.2* SS8, *PtNAC074* SS6, *PtNAC035* SS1, *PtBTF3.1* SS8, *PtBTF3.1* SS10, *PtATAF1.2* SS4, *PtATAF1.1* SS5, *PtNAC052* SS7, *PtNAC028* SS1 and *PtNAC028* SS6. The top of each lane shows the predicted size of the PCR product when splicing occurred at the detected SS. Asterisks indicate bands which were consistent with prediction; (**c**) Distribution of DRSs flanking canonical and non-canonical SSs; (**d**) Alignment of *PtBTF3.2* and its sequencing reads covered SS8. DRSs flanking SS8 are marked in red; (**e**) Model explaining the generation of DRS-mega-primers and how artifacts were amplified by DRS-mega-primers.

To verify poorly covered SSs detected by 3′-RACE-seq, ten canonical SSs with an average coverage of 0.009–0.27% and ten non-canonical SSs with an average coverage of 0.001–0.45% were randomly chosen for verification by RT-PCR (Figure S1, Table S2). Forward and reverse primers were designed within exons flanking the SS and to amplify small PCR products of 100–200 bp (Figure S1, Tables S2 and S5). All 10 canonical SSs were verified with RT-PCR, with predicted molecular sizes and clone sequencing (Table S6); however, for the tested non-canonical SSs, no PCR products were detected with the predicted molecular sizes (Figure 3a,b).

Further analysis of the sequence flanking SSs showed that ~90% (26/29) of the non-canonical SSs possessed direct repetitive sequences (DRSs) with length ≥4 bp, while almost 26% (19/72) of the canonical SSs had DRSs. Furthermore, the long DRSs (6–10 bp) were more prevalent (~51%, 15/29) in the non-canonical SSs, while long DRSs were rare (only ~4%, 3/72) in canonical SSs (Figure 3c,d, Table S2). Thus, we speculated that many non-canonical SSs were derived from PCR artifacts produced from DRS-mega-primers which can be generated from incomplete extension products ending at DRS regions on occasion (Figure 3e). By extension with out-of-register annealing DRS-mega-primers, DNA fragments between two DRSs were skipped, leading to false-positive detection of SSs by deep sequencing. Due to the low amplification efficiency of one-sided specific primers and two rounds of PCR, these DRS-mega-primers and consequent PCR artifacts are more likely to have accumulated in 3′-RACE experiments. In contrast, RT-PCR, using two-sided specific primers and aiming to generate small products can dramatically reduce such artifacts, clearly distinguishing between authentic and artifactual SSs (Figure 3a,b).

### 2.4. Complex AS Events in 14 NAC Transcription Factor Genes

**Figure 4:**
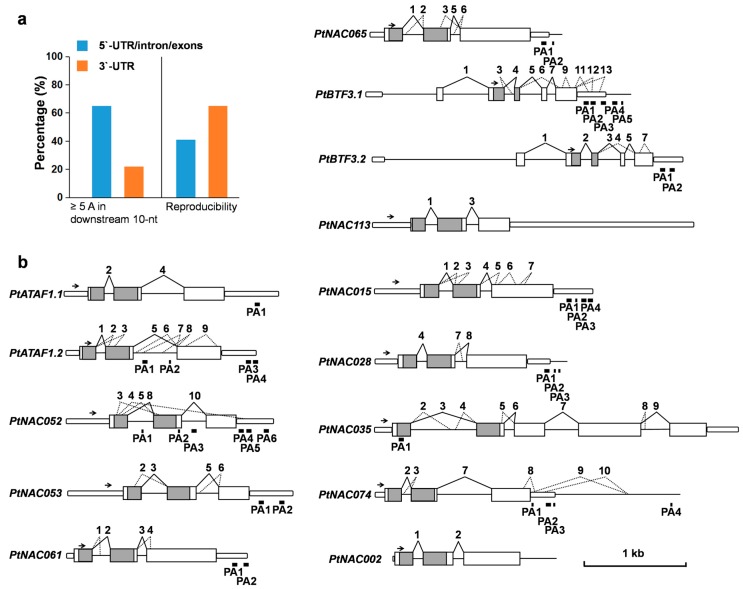
Polyadenylation sites (PASs) and splicing sites of 14 NAC transcription factor genes revealed by 3′-RACE-seq. (**a**) The percentage of ≥5 As (adenosines) in a downstream 10 nt window and reproducibility for candidate PASs; (**b**) Schematic representation of splicing sites and PASs. Rectangle: annotated exon; round rectangle: 5′-UTR or 3′-UTR; straight line: annotated intron; grey rectangle: region encoding NAC domain; zigzag full line: well-covered SS; zigzag broken line: poorly covered SS; tops of zigzag lines are number of each SS, which is discontinuous because of removal of non-canonical sites; short and long bold full line below indicates individual PASs and polyadenylation site clusters (PACs), respectively; arrow: position of 3′-RACE inner primers.

Due to distinctive GT-AG rules and rare long DRSs in flanking sequences, both well- or poorly covered canonical SSs were considered to be authentic ones. Therefore, a total of 72 canonical SSs (32 well-covered and 40 poorly covered SSs) were used to analyze AS events in the 14 transcription factor genes. Based on primary and alternative gene models annotated by *Populus trichocarpa* v3.0 (Phytozome, https://phytozome.jgi.doe.gov/pz/portal.html), 34 poorly covered SSs were newly discovered. Among the 48 annotated SSs within the region downstream of gene-specific 3′ inner primers, 36 (75%) were verified. In the *P. trichocarpa* seedlings under normal growth conditions, SSs of 12 primary gene models (Model 1) were well covered, but for *PtBTF3.1* and *PtNAC113*, the specific SSs of an alternative gene model (Model 2) were dominant. By comparison to the 12 Model 1 and two Model 2, AS events, including alternative 3′ splice site (A3′SS), alternative 5′ splice site (A5′SS), alternative 5′ and 3′ splice site (A5′/A3′SS), exon skipping (ES), and exonic introns (Exitron, EI) [19], were analyzed (Figure 4b, Table S2).

Twelve A3′SSs, 12 A5′SSs and two A5′/A3′SSs were detected with 3′-RACE-seq. Among them, 12 AS sites located in the 5′ regions of genes could introduce premature termination codons (PTCs) or insert/delete amino acids in NAC domains, potentially leading to nonsense-mediated decay (NMD) or alteration of DNA-binding specificity. In addition, three ES-type AS events were detected, which led to removal of most of the coding regions. Eleven EIs of five genes were identified in the last and longest annotated exons. Four EIs had multiples of three nucleotides (EIx3) and splicing resulted in internally deleted proteins. In contrast, seven EIs were non-EIx3 and splicing led to frameshift mutations downstream of the SSs.

### 2.5. Identification of PASs by Removal of Possible Internal Priming Sites

In order to identify PASs, reads with 3′-RACE adaptors were selected and trimmed. In total, 284 and 296 candidate PASs were detected in 5′-UTR/intron/exons and 3′-UTRs, respectively. In total, 65% (185/284) and 22% (65/296) candidate PASs of 5′-UTR/intron/exons and 3′-UTR, respectively, had ≥5 adenosines in a downstream 10-nt window (Figure 4a, Tables S3 and S4). The ratio difference between 5′-UTR/intron/exons (65%) and 3′-UTR (22%) suggested that the effect of internal priming in 5′-UTR/intron/exons was more obvious, compared to those in 3′-UTR. Because the lengths of native poly(A) tails (average 51 nt in *Arabidopsis*) [20] were longer than those within transcripts (maximum 12 nt in 14 target genes), reverse transcription should be more reproducible from native poly(A) tails than from internal A-rich regions. Candidate PAS counting showed 41% (116/284) and 65% (191/296) reproducibility in 5′-UTR/intron/exons and 3′-UTR, respectively. Such reproducibility also suggested a greater degree of internal priming in 5′-UTR/intron/exons than in 3′-UTR.

Internal priming is the foremost challenge for reverse transcriptase-dependent PAS identification [12]. Would the PASs without any internal priming features be considered to be authentic ones? It is well known that plant mRNA 3′-ends exhibit extreme heterogeneity [13,14,15]. Thus, we hypothesized that the PASs without any internal priming features could serve as markers for PAS-rich regions in plants. This hypothesis could be useful for detecting rare and potentially important PASs in 5′-UTR/intron/exons.

To screen out the markers representing PAS-rich regions, we established stringent criteria for eliminating any potential internal priming sites in 5′-UTR/intron/exons. These criteria included: (1) the 3′-stretch immediately downstream of candidate PASs should contain ≤1 adenosine; (2) the 10-nt window downstream of candidate PASs should contain ≤3 adenosines; (3) the 5-nt window upstream and downstream of candidate PASs should not have any other candidate PASs that do not meet criteria (1) or (2); and (4) candidate PASs should be repeatedly detected by independent experiments. It is noteworthy that adding criterion (3) can avoid false positives derived from the sliding of primers. Based on these criteria, we identified two internal exons and four intronic PASs/PACs (PASs within 50 nt of each other were defined as a PAC), which were present in three target genes, that is, *PtNAC052*, *PtATAF1.2* and *PtNAC035* (Figure 4b, Table S3).

Due to the lower disturbance of internal priming, 3′-UTR candidate PASs were screened by less-stringent criteria, as follows: (1) the 3′-stretch immediately downstream of candidate PASs should contain ≤3 adenosines; (2) the 10-nt window downstream of candidate PASs should contain ≤4 adenosines; and (3) candidate PASs should be repeatedly detected by independent experiment. Based on these criteria, 30 3′-UTR PASs/PACs were identified in 11 genes, and between two and five tandem 3′-UTR APAs were revealed in ten genes (Figure 4b, Table S3).

### 2.6. APA Transcripts Were Differentially Regulated across Tissues and in Response to Plant Hormones and Elicitors

**Figure 5:**
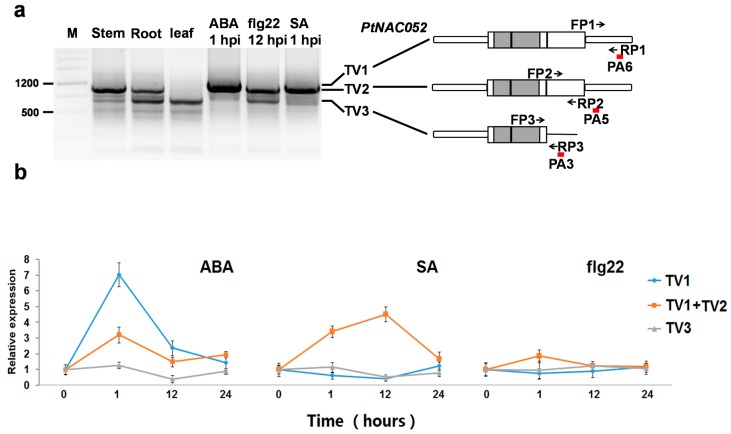
Alternative polyadenylation (APA) transcripts of *PtNAC052* differentially regulated across tissues and in response to plant hormones and elicitors. (**a**) Agarose gel electrophoresis of *PtNAC052* 3′-RACE with cDNA from different tissues and treatments and principle of real-time PCR primers design for transcript variants of *PtNAC052*. Arrows indicate forward primer (FP) and reverse primer (RP). Due to the overlapping of TV1 and TV2, FP2 and RP2 of *PtNAC052* were designed to amplify TV1 and TV2 simultaneously. Rectangle: annotated exon; round rectangle: 5′-UTR or 3′-UTR; straight line: annotated intron; grey rectangle: region encoding NAC domain; short and long bold full line below indicates individual PASs and PACs, respectively; (**b**) Expression patterns of *PtNAC052* TVs during response to ABA, SA and flg22. Expression levels were calculated relative to 0 hpi samples. Bars indicate mean expression ± SD (standard deviation) of three replicates. These experiments were repeated three times with similar results.

As an important regulatory mechanism, APA should respond to endogenous and exogenous cues. The feature of differential regulation of APA transcripts can be regarded as strong evidence for the authenticity of PASs. To verify the candidate PASs, 3′-RACE experiments were performed with cDNA from poplar stems, roots and leaves, and from leaves treated with abscisic acid (ABA) 1 hours post-inoculation (hpi), salicylic acid (SA) 1 hpi or flg22 12 hpi. By clone sequencing of polymorphic bands, different PAS choices were detected in the different cDNA samples of *PtNAC052* (Figure 5a), but were not detected in the samples of the other 13 tested genes (Figure S2).

Clone sequencing of the three major bands of *PtNAC052* showed that the largest band (transcript variant 1; TV1) represented transcripts that ended in 3′-UTR (at 1977 nt of gDNA in PA6). The band next to the largest one (TV2), also represented transcripts that ended in the 3′-UTR (at 1775 nt and 1787 nt in PA5). The small band (TV3) represented transcripts that ended in the second intron (at 1232 nt in PA3, and at 1276 nt adjacent to PA3) (Figure 5a). In addition, the largest band (TV1) was detected exclusively in the ABA-treated leaves, suggested a stress-related 3′-UTR extension [21].

To confirm the differential regulation of APA transcripts, real-time PCR was performed with transcript-variant specific primers. No specific sequences of *PtNAC052* TV2 were distinguishable from those of TV3, so one pair of primers was used for analyzing the mixed expression patterns of TV1 and TV2. Figure 5b illustrates the different expression patterns of *PtNAC052* transcript variants. Upon ABA inoculation, *PtNAC052* TV1 was markedly induced at 1 hpi, reaching 7.05 times the level of 0 hpi samples, and then declined at both 12 and 24 hpi. *PtNAC052* TV1 + TV2 were also induced at 1 hpi, but their expression level (3.21 times the level of 0 hpi samples) was lower than that of TV1. *PtNAC052* TV3 maintained a stable level of expression (similar to 0 hpi) at 1 hpi, with the expression level declining at 12 hpi. Following SA treatment, expression of *PtNAC052* TV1 + TV2 was up-regulated at 1 and 12 hpi, whereas *PtNAC052* TV1 and TV3 expression remained stable or was down-regulated at this stage, suggesting that only *PtNAC052* TV2 was induced. As for flg22 treatment, no *PtNAC052* TVs showed significant up- or down-regulation in response. 

Together, these results, showing differential regulation of *PtNAC052* transcript variants, confirmed the reliability of PAS-rich markers identified by 3′-RACE-seq.

## 3. Discussion

3′-RACE is a classical method to obtain 3′-ends of unknown cDNA sequences adjacent to known sequences, and to determine PASs of mRNA [22,23]. Our initial idea was to conduct 3′-RACE with primers from the 5′-end of genes, followed by HTS, to analyze AS and APA events on most regions of target genes simultaneously. Through detailed analysis of the 3′-RACE-seq results, we gained new insight into traditional 3′-RACE experiments. Historically, 3′-RACE has been known to generate a high background of non-specific and truncated products [24]. The extent of such a background was estimated by 3′-RACE-seq. In 24 3′-RACE experiments, more than half of the sequencing reads were not mapped to target genes, and >30% of the sequencing reads were mapped to other regions of the poplar genome (Table S1), while many internal priming events were indicated in 5′-UTR/intron/exons. The significant Monte Carlo effects between 3′-RACE-seq Experiment 1 and Experiment 2 suggested that two or more independent technical replicates were required to determine low-abundance SSs, using DNA deep sequencing of PCR products. By deep sequencing, we also discovered a new pitfall in 3′-RACE experiments, namely, the production of artifacts caused by DRS-mega-primers. This pitfall could be intrinsic to 3′-RACE, because the low-efficiency amplification conducted by one-sided gene-specific primers is likely to lead to many incomplete amplification products stopping at DRSs, while two-round PCRs with >70 cycles may provide more opportunity for the emergence and accumulation of artifacts. These low-abundance artifacts (0.001–0.6% of the percentage of coverage in this study) could do no harm for traditional 3′-RACE experiments which rely on low-throughput clone sequencing, but could seriously disturb the SS analysis of high-throughput sequencing.

Artifacts produced by DRS-mega-primers usually lead to identification of non-canonical SSs, which are rare in plants [7,8,25]. Therefore, taking only canonical SSs into account would be a convenient criterion for large-scale screening. By deep sequencing of a limited region, many poorly covered canonical SSs were revealed; for example, among 0.72 million reads mapped to *PtNAC053*, we detected 21 reads that spanned SS2. This kind of poorly covered SSs can barely be detected by transcriptome-wide HTS analysis under present sequencing depth in plants, of which the average coverage is usually <50,000 reads per gene [7,8,10]. In contrast to the other target strategy, RT-PCR-seq, which can only analyze SSs which are adjacent to known SSs, the 3′-RACE-seq method described here can detect SSs without any previous cues, such as novel EIs in annotated exons and novel ESs spanning a large region. This feature of 3′-RACE-seq also indicates its potential application for other eukaryotes with limited sequence information.

APA events in plants are known to be involved in plant flowering, dormancy and stress, but only a few have been well studied in plants [26,27,28,29,30]. This is partly because high-throughput transcriptome-wide PASs analyses have been restricted to model plant species, such as *Arabidopsis thaliana*, *Medicago truncatula* and *Oryza sativa*, and are disturbed by internal priming [11,13,31,32], whereas low-throughput 3′-RACE of genes and gene families are disturbed by both internal priming and non-specific amplification. In this study, we hypothesized that PASs without any internal priming features could serve as markers for heterogeneous 3′-ends in plants, and that extremely high sequencing depth of 3′-RACE products could detect such markers. A subsequent series of experiments confirmed this hypothesis. 3′-RACE-seq resulted in the production of many candidate PASs. Using very stringent criteria, three genes were screened out that possessed intronic/internal exon PASs. By using 3′-RACE and real-time PCR, one gene was confirmed to have tightly regulated APA transcripts, which ended in/around the previously identified PASs. This indicates that 3′-RACE-seq can accurately identify those gene regions which are subjected to APA.

## 4. Materials and Methods 

### 4.1. Plant Material and Plant Hormone and Elicitor Treatments

Sterile seedlings of *P. trichocarpa* ‘Nisqually-1′ were used for 3′-RACE-seq. The sterilized seedlings were grown on Murashige and Skoog medium (pH 5.6), supplemented with 3% sucrose, 0.3% (*w*/*v*) Gelrite and 5 g/L activated charcoal, under a cycle of 16 h light/8 h dark with temperatures set at 22 °C/18 °C (day/night) [33], for 4 weeks. Abscisic acid (ABA), the flagellin fragment peptide flg22 (TRLSSGLKINSAKDDAAGLQIA from Pseudomonas syringae DC3000, >90% purity) (GenScript, Nanjing, China) [34] and salicylic acid (SA) were diluted to 0.01 mmol/L, 2 μmol/L and 5 mmol/L, respectively, with Milli-Q water containing 0.01% Silwet L-77. The solutions were sprayed onto the leaves, then the treated leaves were harvested at 0, 1, 12 and 24 h post-inoculation (hpi), immediately snap-frozen in liquid nitrogen, and stored at −70 °C. 

### 4.2. 3′-RACE Experiment

Total RNA was extracted using the RNAprep Pure Plant Kit (Tiangen, Beijing, China) with on-column DNaseI digestion. According to the sequences of annotated gene models of Phytozome (http://phytozome.jgi.doe.gov/) *P. trichocarpa* v3.0, 12 pairs of 3′-RACE primers were designed for 14 NAC transcription factor genes (Table S5). The outer and inner primers were adjacent to the initiation codon. cDNA was synthesized from 1 μg total RNA using a 3′-RACE adaptor (5′-GCGAGCACAGAATTAATACGACTCACTATAGGT12VN-3′) with the Promega ImPromII Reverse Transcription System (Promega, Madison, WI, USA). cDNA was then diluted 1:4 with nuclease-free water. PCR was performed using KOD-plus-Neo DNA polymerase (Toyobo, Osaka, Japan) in a 50 μL reaction volume. Two independent 3′-RACEs’ groups, each of which was composed of twelve 3′-RACE experiments, were amplified by the PCR with either 2 μL starting template and 35 cycles per round (Experiment 1), or 4 μL starting template and 33 cycles per round (Experiment 2). PCR products of each group were pooled as one sample, separately, resulting in two samples (Experiment 1 and Experiment 2). Pooled PCR products (500 μL) were purified using 1% agarose gel electrophoresis to remove primer dimers. The target band was excised and isolated from the gel, using the DNA Gel Extraction Kit (Tiangen, Beijing, China).

### 4.3. HTS and Data Analysis

Purified PCR products from Experiment 1 and Experiment 2 were sheared by sonication to generate two DNA fragment libraries of 150–600 bp. The fragmented samples were purified through QIAquick PCR cleanup columns (Qiagen, Hilden, Germany) to build libraries using a TruSeq DNA Sample Prep Kit (Illumina, San Diego, CA, USA). The TruSeq libraries were sequenced on Illumina HiSeq2000 with 125 bp paired-end reads by the Annoroad Gene Technology Corporation (Beijing, China). Each library produced about 10 million raw reads. Low-quality bases (≤Q30), adaptors and short reads (≤50 bp) were removed. The reads (2 × 125 bp paired-end) were mapped against the *P. trichocarpa* genome v3.0 (https://phytozome.jgi.doe.gov/pz/portal.html) using TopHat (v2.0.12, https://ccb.jhu.edu/software/tophat/index.shtml) with a maximum of two mismatches [35]. Converting of SAM to BAM file was done using SAMtools (v1.3.1, http://www.htslib.org/). Depth of reads in poplar genome was calculated by ‘depth’ command in SAMtools.

The mapping results were viewed with NGS (next generation sequencing) alignment viewer Tablet [36]. All predicted splice sites were manually checked using Tablet. Reads with more than one mismatch around the splice sites were removed. The SSs covered by ≥10 reads in each experiment were selected for detection of splice sites.

On the other hand, the reads were filtered with in-house Perl script for PAS analysis. The remaining reads were aligned against *P. trichocarpa* genome v3.0, and PASs were counted with Tablet. Any site represented by ≥5 reads in each experiment was chosen as a candidate PAS for further analysis.

### 4.4. Real-Time PCR

RNA extraction and reverse transcription methods were the same as for the 3′-RACE experiment, except that cDNA was synthesized with oligo(dT)_18_. According to the distinguishable region of different transcript variants, specific primers were designed for TV1/2/3 of *PtNAC052* (Table S5). Primer specificity was tested by BLAST against the Poplar genome database and experimentally confirmed (Figure S3). *Elongation factor 1-a* was chosen as the internal control, as previously described [37]. The expression levels from samples at 0 hpi were set to 1 and all subsequent sample expression levels were compared to the 0 hpi samples. PCR was carried out in a 20-μL reaction system using the FastStart Universal SYBR Green Master Mix (Roche, Penzberg, Germany). Real-time PCR was performed on an Applied Biosystems 7500 Real-time PCR System (Applied Biosystems, Waltham, MA, USA). The following cycling conditions were used: 50 °C for 2 min, 95 °C for 10 min, and 40 cycles of 95 °C for 15 s and 60 °C for 1 min. These experiments were repeated three times with independently inoculated samples. The comparative C_T_ method was used for analyzing real-time PCR data [38].

### 4.5. Availability of Supporting Data

Illumina short reads for two 3′-RACE experiments of fourteen NAC transcription factor genes in *Populus* have been deposited in the NCBI SRA (the Sequence Read Archive) database under BioProject PRJNA329448.

## 5. Conclusions

In this study, we explored a 3′-RACE-seq method to simultaneously identify splice and polyadenylation sites in plants. By in-depth sequencing of limited regions, this method exhibited sensitivity, but our analysis also indicated that the results from this approach must be used with care. Firstly, non-canonical and long DRS-flanking SSs could result from PCR artifacts. Secondly, due to the heavy influence of internal priming, candidate PASs must be filtered, using stringent criteria. However, this target HTS approach can robustly discover active post-transcriptional regulation cues that merely rely on only limited sequence information. In addition, small-scale HTS could be performed at low-cost, due to the low amount of sequencing data required. As a result, 3′-RACE-seq seems suitable for small-scale AS and APA research in the initial stage.

## Supplementary Materials

The following are available online. Figure S1: The position of forward and reverse primers, Figure S2: Agarose electrophoresis of 3¢-RACE with cDNA of different tissue and treatments, Figure S3: Agarose electrophoresis of PCR products to confirm real-time PCR primer specificity, Table S1: Statistical data of 3¢-RACE-seq, Table S2: Summary of all detected SSs, Table S3: Summary of PASs that meet filter criteria, Table S4: Counting results of candidate PASs, Table S5: Primers used in experiments, Table S6: Clone sequencing data of 10 canonical SSs verified by RT-PCR.
