# Supplementary material for: Capturing the Alternative Cleavage and Polyadenylation Sites of 14 NAC Genes in Populus Using a Combination of 3′-RACE and High-Throughput Sequencing
Source: Molecules. 2018 Mar 8;23(3):608. doi: 10.3390/molecules23030608 (PMC6017670; doi:10.3390/molecules23030608)
Supplement: Supplementary file 1 [file molecules-23-00608-s001.docx]

**Capturing the alternative cleavage and polyadenylation sites of 14 NAC genes in *Populus* using a combination of 3′-RACE and high-throughput sequencing**

Haoran Wang^1^, Mingxiu Wang^1^*, Qiang Cheng^1^*

^1^The Southern Modern Forestry Collaborative Innovation Center, Nanjing Forestry University, Nanjing, Jiangsu, 210037, China

*Corresponding author: Dr. Mingxiu Wang, The Southern Modern Forestry Collaborative Innovation Center, Nanjing Forestry University, Nanjing, Jiangsu, 210037, China Email: **[mxwang@njfu.edu.cn](mailto:chengqiang@njfu.edu.cn)**

*Corresponding author: Dr. Qiang Cheng, The Southern Modern Forestry Collaborative Innovation Center, Nanjing Forestry University, Nanjing, Jiangsu, 210037, China Email: **[chengqiang@njfu.edu.cn](mailto:chengqiang@njfu.edu.cn)**


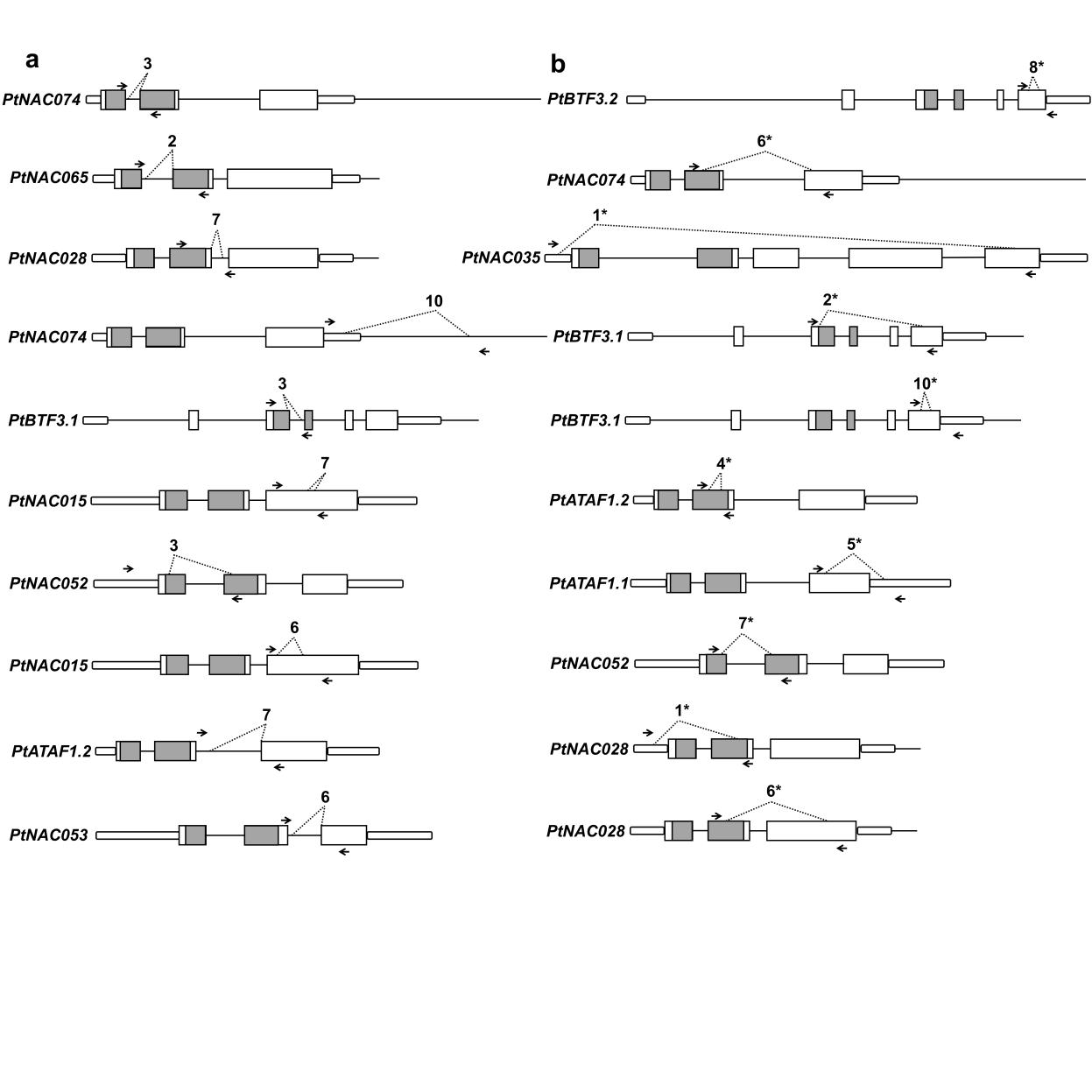


**Fig. S1** The position of forward and reverse primers. (a) Primers to test 10 canonical SSs. (b) Primers to test 10 non-canonical SSs. Arrows indicate forward and reverse primers. Zigzag broken line, target poorly covered SS.


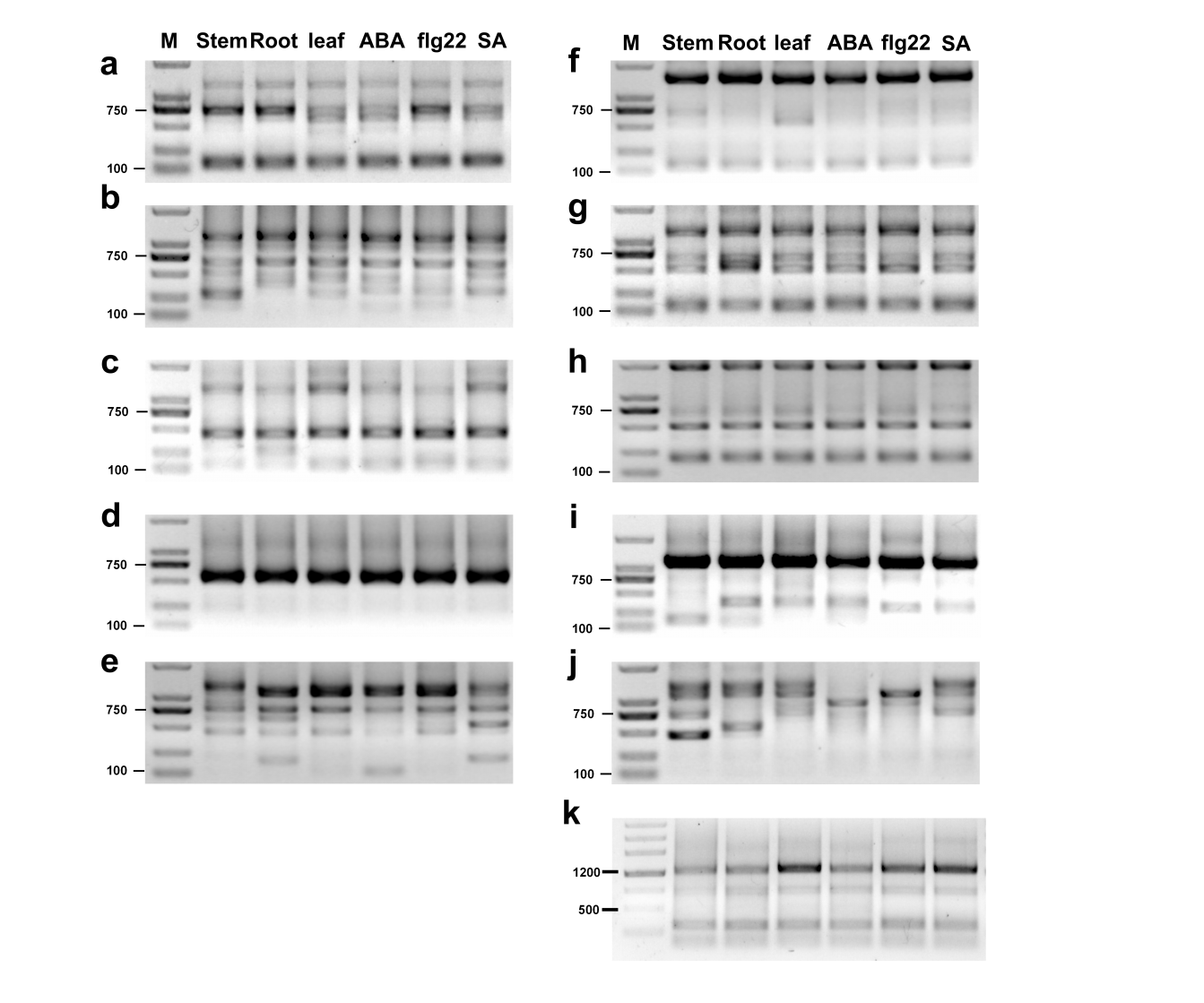


**Fig. S2** Agarose electrophoresis of 3′-RACE with cDNA of different tissue and treatments. **(a)** *PtATAF1.1* **(b)** *PtNAC053* **(c)** *PtNAC061* and *PtNAC065* **(d)** *PtBTF3.1* and *PtBTF3.2* **(e)** *PtNAC113* **(f)** *PtNAC015* **(g)** *PtNAC028* **(h)** *PtNAC035* **(i)** *PtNAC074* **(j)** *PtNAC002* **(k)** *PtATAF1.2*. lane 1, DNA ladder; lane 2, cDNA of stems; lane 3, cDNA of roots; lane 4, cDNA of leaves; lane 5, cDNA of ABA (1 hpi) treated leaves; lane 6, cDNA of flg22 (12 hpi) treated leaves; lane 7, cDNA of SA (1 hpi) treated leaves. Although the presentation of polymorphism of DNA bands in *PtNAC113*, *PtNAC002*, *PtNAC074* and *PtNAC053*, the cloning and sequencing results showed that they are unspecific products.


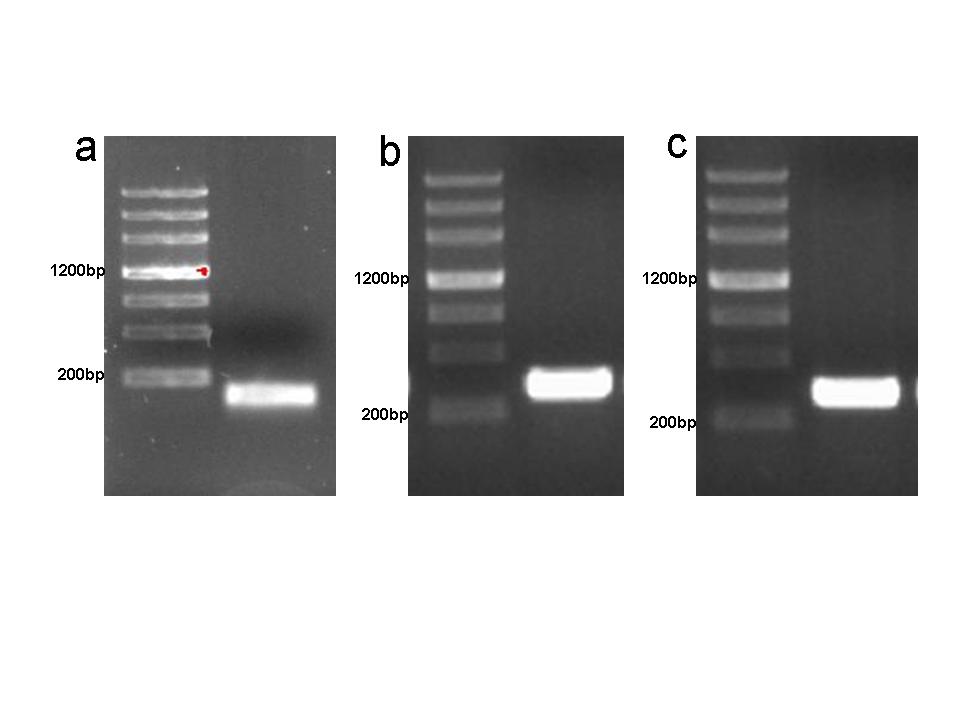


**Fig. S3** Agarose electrophoresis of PCR products to confirm real-time PCR primer specificity. **(a)**1.5% agarose electrophoresis of PCR products to confirm PtNAC052-TV1 qPCR-primers. **(b)**1% agarose electrophoresis of PCR products to confirm PtNAC052-TV2 qPCR-primers. **(c)**1% agarose electrophoresis of PCR products to confirm PtNAC052-TV3 qPCR-primers. lane 1, DNA ladder; lane 2, PCR products with cDNA template mixed of (leaves, ABA (1 hpi) treated leaves, flg22 (12 hpi) treated leaves, SA (1 hpi) treated leaves) and 40 cycles’ amplification.

**Table S1** Statistical data of 3′-RACE-seq

| Experiment | Total reads | Reads mapped to poplar genome | Reads mapped to target genes | Mapping ratio of genome | Mapping ratio of target genes |
| --- | --- | --- | --- | --- | --- |
| Experiment 1 | 9,386,612 | 6,660,605 | 3,432,262 | 70.9% | 36.6% |
| Experiment 2 | 8,904,012 | 6,427,833 | 3,836,569 | 72.2% | 43.1% |

**Table S2** Summary of all detected SSs

| Gene ID/Gene name^a^ | SSs percentage^b^ | Reads： Experiment 1/Experiment 2 | AS type^d^ of each SSs | DRS^e^ | Gene model of SSs^f^ |
| --- | --- | --- | --- | --- | --- |
|  | SS^c^: Experiment 1/  Experiment 2 |  |  |  |  |
| Potri.005G180200.1 PtATAF1.1 | SS1^*^: 0.003%/0%  SS2: 20%/22.2%  SS3^*^: 0.006%/0%  SS4: 4.92%/5.58%  SS5^*^: 0.004%/0% | 10/0  62,614/56,942  19/0  15,407/14,272  11/0 | Annotated  Annotated | ACAGCAGGC  GAAAG | Model 1  Model 1 |
| Potri.002G081000.1 PtATAF1.2 | SS1: 21%/18%  SS2: 0.01%/0.07%  SS3: 0.01%/0.07%  SS4^*^: 0.07%/0%  SS5: 9.4%/10%  SS6: 0.3%/0.3%  SS7: 0.02%/0.01%  SS8: 0.004%/0.002%  SS9: 0%/0.1% | 65,443/76,050 37/281  29/307  232/0  29,436/43,305  943/1198  66/52  11/10  0/441 | Annotated  A3`SS  A3`SS  Annotated  A5`SS  A5`SS  A5`SS  EI | AGGGAG | Model 1  Novel  Novel  Model 1  Model 2  Novel  Novel  Novel |
| Potri.003G166500.1 PtNAC052 | SS1^*^: 0.004%/0%  SS2^*^: 0.002%/0%  SS3：0.018%/0%  SS4: 0.015%/0%  SS5: 0.005%/0.01%  SS6^*^: 0.001%/0%  SS7^*^: 0.003%/0%  SS8: 14%/15.4%  SS9^*^: 0.24%/0%  SS10: 9.32%/10.7%  SS11^*^ :0.047%/0% | 28/0  15/0  122/0  104/0  34/144  10/0  23/0  97,030/64,814  1,629/0  64,561/44,936  328/0 | A5` and 3` SS  SE, A5`and 3`SS  A5`SS  Annotated  Annotated | AAGGCA  GGAGA  TTAGGTT  AGGTG  CAAGT  CAGGT  GGAAGG  CAGG  AAGAAAACA | Novel  Novel  Novel  Model 1  Model 1 |
| Potri.001G061200.1 PtNAC053 | SS1^*^: 0%/0.033%  SS2: 0.003%/0.003%  SS3: 13.5%/14.5%  SS4^*^: 0%/0.21%  SS5: 6.43%/5.77%  SS6: 0.013%/0.053% | 0/122  10/11  49,769/53,349  0/765  23,666/21,279  49/197 | A5`SS  Annotated  Annotated  A5`SS | TTAAG  AGGTG  CAGGT  GAAG  CAGG | Novel  Model 1  Model 1  Novel |
| Potri.002G178700.1 PtNAC061 | SS1: 0.018%/0%  SS2: 22.1%/26.1%  SS3: 11.5%/14.9%  SS4: 0.012%/0% | 20/0  24,593/42,483  12,811/24,344  14/0 | A3`SS  Annotated  Annotated  A3`SS |  | Novel  Model 1  Model 1  Novel |
| Potri.014G104800.1  PtNAC065 | SS1: 28.4%/33.9%  SS2: 0.027%/0%  SS3: 0.33%/0%  SS4^*^: 0%/0.15%  SS5: 13.4%/15.8%  SS6: 0.022%/0% | 48,048/88,836  46/0  553/0  0/398  22,747/41,363  37/0 | Annotated  A5`SS  A5`SS  Annotated  A5`SS | GTCT  TATAG  AGGT | Model 1  Novel  Novel  Model 1  Novel |
| Potri.015G029800.2 PtBTF3.1 | SS1: 0.25%/0.019%  SS2^*^: 0.0135%/0%  SS3: 0.037%/0.033%  SS4: 40.83%/43.29%  SS5: 42.64%/43.74%  SS6: 0.009%/0.006%  SS7: 31.14%/30.81%  SS8^*^: 0.08%/0%  SS9: 0.005%/0%  SS10^*^: 0.60%/0.31%  SS11: 0.025%/0.027%  SS12：0.006%/0.006%  SS13: 0%/0.007% | 590/59  32/0  88/104  96,620/134,942  100,909/136,355  21/18  73,709/96,053  190/0  11/0  1,420/963  58/84  14/19  0/21 | Annotated  A3`SS  Annotated  Annotated  SE and A3`SS  Annotated  EI  EI  EI  EI | GAAGG  AGTCTAC  CAGA  GCAG  CTGCAGCAGA | Model 1  Novel  Model 2  Model 1  Novel  Model 1  Novel  Novel  Novel  Novel |
| Potri.012G037900.1  PtBTF3.2 | SS1: 0.15%/0.014%  SS2: 46.6%/43%  SS3: 45%/44.6%  SS4: 0.009%/0%  SS5: 28.4%/28%  SS6^*^: 0%/0.01%  SS7: 0%/0.11%  SS8^*^: 0.07%/0.03% | 214/25  61,401/75,646  64,806/78,391  13/0  40,875/49,248  0/19  0/191  105/53 | Annotated  Annotated  Annotated  SE  Annotated  EI | TGCAG  TAGGT  AGCA  TGCTGC  GCAGA | Model 1  Model 1  Model 1  Novel  Model 1  Novel |
| Potri.011G046700.1 PtNAC002 | SS1: 13.4%/20.7%  SS2: 11.7%/11.1% | 19,678/10,282  17,092/5,511 | Annotated  Annotated | GTAAG | Model 1  Model1 |
| Potri.012G001400.1 PtNAC015 | SS1: 12.2%/10.3%  SS2: 0.26%/0.28%  SS3: 0.004%/0.017%  SS4: 8.34%/8.86%  SS5: 0.12%/0.10%  SS6：0%/0.24%  SS7: 0.27%/0.27% | 45,954/43,622  985/1207  16/70  31,465/37,541  437/437  0/1015  1,008/1,164 | Annotated  A3`SS  A3`SS  Annotated  A3`SS  EI  EI |  | Model 1  Model 2  Novel  Model 1  Novel  Novel  Model 3 |
| Potri.017G086200.1 PtNAC028 | SS1^*^: 0%/0.002%  SS2^*^: 0.003%/0%  SS3^*^: 0.038%/0%  SS4: 17.8%/18.6%  SS5^*^: 0.008%/0%  SS6^*^: 0.028%/0%  SS7: 0.076%/0.056%  SS8: 7.09%/7.09% | 0/11  10/0  146/0  68,303/82,215  31/0  109/0  293/249  27,260/31,332 | Annotated  A3’SS  Annotated | AAGA  ATTTTTTTT  GAAAACA  AGGAC  CAAGGA | Model 1  Novel  Model 1 |
| Potri.005G200100.1 PtNAC035 | SS1^*^: 0.09%/0.028%  SS2: 0%/0.004%  SS3: 13.2%/10.8%  SS4: 0%/0.008%  SS5: 0.033%/0%  SS6: 3.75%/4.37%  SS7: 3.42%/3.92%  SS8: 0%/0.008%  SS9: 3.29%/3.90% | 104/90  0/12  15,319/34,451  0/27  39/0  4,337/13,930  3,958/12,506  0/24  3,809/12,423 | A3`SS  Annotated  A5`SS  A5`SS  Annotated  Annotated  A3`SS  Annotated | GTCAAAAA  CAGG  CAGGTT  CAGGT | Novel  Model 1  Novel  Novel  Model 1  Model 1  Novel  Model 1 |
| Potri.004G181900.1 PtNAC074 | SS1^*^: 0%/0.02%  SS2: 11.15%/10.81%  SS3: 0.047%/0.037%  SS4^*^: 0.27%/0%  SS5^*^: 0%/0.19%  SS6^*^: 0.34%/0%  SS7: 12.2%/13.1%  SS8: 0.013%/0.057%  SS9: 0.016%/0%  SS10: 0.045%/0.01% | 0/114  28,315/55,282  120/191  681/0  0/949  864/0  31,059/67,186  35/293  42/0  115/55 | Annotated  A5`SS  Annotated  EI  EI  EI | TATC  CAGGT  GGAAG  GATTCTGGAAG  TGGAAG | Model 1  Novel  Model 1  Model 2  Novel  Novel |
| Potri.009G019200.2 PtNAC113 | SS1: 14.9%/10%  SS2*: 0%/0.38%  SS3: 9.06%/8.79% | 27,420/12,809  0/492  16,651/11,259 | Annotated  Annotated | TTATGT | Model 1  Model 1 |

a, according to Phytozome Populus trichocarpa v3.0

b, number of reads spanned intron/ number of reads covered target gene×100%

c, SSs were numbered from 5′ terminus to 3′ terminus

d, A3′ SS is alternative 3′ splice site; A5′ SS is alternative 5′ splice site; SE is exon skipping; EI (Exitron) is intron locating in annotated exon.

e, direct repetitive sequences flanking SSs

f, Model 1, primary gene model; Model 2 or Model 3, alternative gene model 2 or 3; Novel, newfound SSs in this study.

*, non-canonical SSs

red, Annotated SSs in the region encoding NAC domain

**Table S3** Summary of PASs that meet filter criteria

| Gene ID/Gene name^a^ | Position of each PASs^b^ | Raw reads: Experiment 1/Experiment 2 | Downstream 10-nt sequence | PAC/PAS | APA type |
| --- | --- | --- | --- | --- | --- |
| Potri.005G180200 PtATAF1.1 | 1885  1892  1895  1896  1909 | 371/116  107/31  97/20  860/326  206/186 | AATCTGCACG  ACGTATATCA  TATATCATTT  ATATCATTTG  AATCTGTGTT | PA1 | NA |
| Potri.002G081000 PtATAF1.2 | 760  780 | 39/14  9/8 | ACTTTTTTGG  ACGATAGTTG | PA1 | intronic APA |
|  | 1206 | 28/42 | ATCTCTGGAA | PA2 | intronic APA |
|  | 1786  1789  1805 | 59/45  47/38  89/99 | AGCAGACTTG  AGACTTGCCT  AAACTTATTG | PA3 | tandem 3′UTR APA |
|  | 1850  1859  1860  1861  1862  1863  1864  1865  1866  1869 | 25/53  25/62  299/401  274/336  62/86  64/107  36/48  13/22  9/21  36/42 | AATTTGTGTT  TTTTTTTTTT  TTTTTTTTTT  TTTTTTTTTT  TTTTTTTTTT  TTTTTTTTTT  TTTTTTTTTT  TTTTTTTTTT  TTTTTTTTTT  TTTTTTTTTT | PA4 | tandem 3′UTR APA |
| Potri.003G166500 PtNAC052 | 760 | 15/45 | ATTGCCACGT | PA1 | intronic APA |
|  | 1120 | 13/19 | GCCCTGCTAA | PA2 | internal exon APA |
|  | 1228  1231  1232 | 8/5  30/7  87/68 | ACTGAGATTC  GAGATTCATC  AGATTCATCT | PA3 | intronic APA |
|  | 1706  1710  1725  1726  1727 | 11/26  6/12  7/8  14/13  83/38 | AGCCATTAGT  ATTAGTTCTT  GTATTTAGGT  TATTTAGGTG  ATTTAGGTGT | PA4 | tandem 3′UTR APA |
|  | 1775  1787  1788  1789  1790  1796 | 272/159  58/64  19/33  44/39  169/107  8/8 | AATGCTTGTG  CTTAGGTTTC  TTAGGTTTCG  TAGGTTTCGT  AGGTTTCGTC  CGTCTCTCTC | PA5 | tandem 3′UTR APA |
|  | 1954  1970  1981  1983  1999 | 97/16  6/6  160/33  29/7  30/6 | AATTGATATT  TTTTTGCAAA  AGAAATGTTT  AAATGTTTCT  ATGTTTAAGC | PA6 | tandem 3′UTR APA |
| Potri.001G061200 PtNAC053 | 1883  1888  1900  1908  1909  1910  1911  1913  1914  1915  1919 | 22/11  38/33  65/96  12/85  168/184  22/27  18/22  93/130  10/12  32/52  8/14 | AATCTAATGC  AATGCTTGTG  TTTCGGTTTC  TTCGTCTCTC  CTTCTCTCTG  GTCTCTCTCA  TCTCTCTCAT  TCTCTCATCT  CTCTCATCTG  TCTCATCTGT  ATCTGTGAGT | PA1 | tandem 3′UTR APA |
|  | 2086  2087  2093  2101  2103  2114  2115  2127  2131 | 20/17  112/117  27/6  19/36  74/96  30/30  318/240  28/22  20/9 | TAGTGATATT  AGTGATATTT  ATTTCTCCTC  TCCTTTGCAA  CTTTGCAAAT  GAAATGTTTC  AAATGTTTCA  AAACATGTTT  ATGTTTAAGC | PA2 | tandem 3′UTR APA |
| Potri.002G178700.1 PtNAC061 | 1651  1673 | 27/143  38/24 | ATCAAGATGT  AATTTGTTTT | PA1 | tandem 3′UTR APA |
|  | 1765  1766 | 47/16  43/37 | TTCTCTTGCC  TCTCTTGCCT | PA2 | tandem 3′UTR APA |
| Potri.014G104800 PtNAC065 | 1739  1741  1745  1746 | 38/50  35/44  86/103  23/31 | AGATGCGATG  ATGCGATGAC  GATGACACTG  ATGACACTGT | PA1 | tandem 3′UTR APA |
|  | 1797 | 80/79 | AATTTGTTTT | PA2 | tandem 3′UTR APA |
| Potri.015G029800.2 PtBTF3.1 | 2162  2163  2164  2165  2168  2169  2170  2171  2172  2174  2175  2176  2178  2179  2180  2181  2182  2183  2185  2186  2199 | 20/29  122/238  671/1027  9/9  10/21  38/102  154/258  29/31  244/597  7/22  40/74  135/244  21/62  29/40  24/69  12/25  138/281  126/159  7/23  502/867  49/92 | TTATGTTCTC  TATGTTCTCA  ATGTTCTCAG  TGTTCTCAGT  TCTCAGTGAC  CTCAGTGACT  TCAGTGACTC  CAGTCACTCT  AGTGACTCTT  TGACTCTTTA  GACTCTTTAT  ACTCTTTATG  TCTTTATGAT  CTTTATGATG  TTTATGATGT  TTATGATGTT  TATGATGTTG  ATGATGTTGT  GATGTTGTTG  ATGTTGTTGT  AGAGTTTCGA | PA1 | tandem 3′UTR APA |
|  | 2212  2214  2215  2226  2228  2240  2241  2243  2247  2251  2254 | 69/124  8/16  93/220  41/110  68/74  46/89  19/36  12/13  14/12  98/120  216/457 | ATTACACGCT  TACACGCTTT  ACACGCTTTG  AGAAATTCGT  AAATTCGTGA  GATATTTGAT  ATATTTGATT  ATTTGATTAC  GATTACCAAG  ACCAAGTCTG  AAGTCTGTGC | PA2 | tandem 3′UTR APA |
|  | 2273  2274  2280  2309 | 19/35  71/134  7/16  7/12 | GAATGTCAGC  AATGTCAGCT  AGCTCCATAG  ATTGGAGGTC | PA3 | tandem 3′UTR APA |
|  | 2411  2416  2417  2434 | 81/82  9/10  10/15  60/103 | ACTTGGATTT  GATTTGTGGG  ATTTGTGGGC  ATGCTGGGCT | PA4 | tandem 3′UTR APA |
|  | 2499 | 15/15 | ATAGATTTGA | PA5 | tandem 3′UTR APA |
| Potri.012G037900.1  PtBTF3.2 | 2876  2877  2883  2893  2895 | 24/54  89/124  18/34  76/166  16/16 | TATGTTCTCT  ATGTTCTCTG  TCTGTGACTC  ATAATGGTTG  AATGGTTGTT | PA1 | tandem 3′UTR APA |
|  | 2923  2926  2935  2937  2938  2949  2950  2951  2953  2966 | 50/151  21/32  307/555  17/26  223/308  28/38  218/260  99/238  134/154  17/28 | AATATACTTT  ATACTTTTTC  CTGAAATTCG  GAAATTCGTG  AAATTCGTGA  TGATATTTTC  GATATTTTCC  ATATTTTCCT  ATTTTCCTGG  AATGTCAGCA | PA2 | tandem 3′UTR APA |
| Potri.011G046700 NAC002 | NA | NA | NA | NA | NA |
| Potri.012G001400 NAC015 | 1914  1924  1931  1936  1941  1942  1943  1944  1945  1946  1947  1951  1957  1958  1960 | 21/9  35/15  155/96  103/231  16/13  14/53  264/432  211/399  83/175  72/142  185/174  11/36  17/26  138/146  14/23 | AAATTATCTT  ATGAATTATC  ATCTCTTCCT  TTCCTCCTCC  GGAGGAGGAA  CTCCTCGTCG  TCCTCGTCGT  CCTCGTCGTT  CTCGTCGTTG  TCGTCGTTGT  CGTCGTTGTC  GTTGTCTATA  TATATCTGTT  ATATCTGTTG  ATCTGTTGGG | PA1 | tandem 3′UTR APA |
|  | 2009 | 14/17 | AGAATCTGTC | PA2 | tandem 3′UTR APA |
|  | 2084  2087 | 34/96  69/44 | AATAAGTGCT  AAGTGCTTAG | PA3 | tandem 3′UTR APA |
|  | 2104  2107  2111 | 17/139  23/8  36/36 | ATTATTGAGA  ATTGAGATTA  AGCATAATCT | PA4 | tandem 3′UTR APA |
| Potri.017G086200.1 NAC028 | 1712  1716 | 26/17  9/22 | ACGCAAATTT  AAATTTGTTA | PA1 | tandem 3′UTR APA |
|  | 1773 | 53/62 | AGTTCTTGAG | PA2 | tandem 3′UTR APA |
|  | 1809 | 37/28 | AAGAGTCTGA | PA3 | tandem 3′UTR APA |
| Potri.005G200100.1 NAC035 | 221  224 | 321/32  18/9 | GCCGCCTGGG  GCCTGGGTTC | PA1 | internal exon APA |
| Potri.004G181900.1 NAC074 | 1609 | 29/95 | AGATAACTTC | PA1 | tandem 3′UTR APA |
|  | 1771  1772  1776  1777  1780  1783  1792  1803 | 12/8  344/610  21/58  388/780  95/236  78/25  144/242  18/47 | TAATTTACCA  AATTTACCAT  TACCATTGCT  ACCATTGCTC  ATTGCTCTGA  GCTCTGAATA  AATCAATCCT  AAATACTCCC | PA2 | tandem 3′UTR APA |
|  | 1820 | 79/82 | AAGTGGAATG | PA3 | tandem 3′UTR APA |
|  | 3057 | 12/15 | AAAGTCGTGG | PA4 | tandem 3′UTR APA |
| Potri.009G019200.2 NAC113 | NA | NA | NA | NA | NA |

a, according to Phytozome Populus trichocarpa V3.0

b, the position on gDNA sequence of target genes

**Table S4** Counting results of candidate PASs

| *PtBTF3.1*  3′-UTR Position：2075 | | | |
| --- | --- | --- | --- |
| Position^a^ | 10-nt window | Experiment 1  Reads | Experiment 2  Reads |
| 5′-UTR/intron/exons | | | |
| 1386 | AAAGGTAAAG | 10 | N/A |
| 1536 | AAACTAAGAG | N/A | 10 |
| 1579 | AATGAAAAGT | 9 | N/A |
| 1919* | AAGAAATTGG | 12 | 25 |
| 3′-UTR | | | |
| 2162* | TTATGTTCTC | 20 | 29 |
| 2163* | TATGTTCTCA | 122 | 238 |
| 2164* | ATGTTCTCAG | 671 | 1027 |
| 2165* | TGTTCTCAGT | 9 | 9 |
| 2168* | TCTCAGTGAC | 10 | 21 |
| 2169* | CTCAGTGACT | 38 | 102 |
| 2170* | TCAGTGACTC | 154 | 258 |
| 2171* | CAGTCACTCT | 29 | 31 |
| 2172* | AGTGACTCTT | 244 | 597 |
| 2174* | TGACTCTTTA | 7 | 22 |
| 2175* | GACTCTTTAT | 40 | 74 |
| 2176* | ACTCTTTATG | 135 | 244 |
| 2178* | TCTTTATGAT | 21 | 62 |
| 2179* | CTTTATGATG | 29 | 40 |
| 2180* | TTTATGATGT | 24 | 69 |
| 2181* | TTATGATGTT | 12 | 25 |
| 2182* | TATGATGTTG | 138 | 281 |
| 2183* | ATGATGTTGT | 126 | 159 |
| 2184 | TGATGTTGTT | N/A | 11 |
| 2185* | GATGTTGTTG | 7 | 23 |
| 2186* | ATGTTGTTGT | 502 | 867 |
| 2191 | GTTGTGTTAG | N/A | 7 |
| 2199* | AGAGTTTCGA | 49 | 92 |
| 2212* | ATTACACGCT | 69 | 124 |
| 2213 | TTACACGCTT | N/A | 6 |
| 2214* | TACACGCTTT | 8 | 16 |
| 2215* | ACACGCTTTG | 93 | 220 |
| 2217 | ACGCTTTGCA | N/A | 6 |
| 2223 | TGCAGAAATT | N/A | 8 |
| 2226* | AGAAATTCGT | 41 | 110 |
| 2228* | AAATTCGTGA | 68 | 74 |
| 2239 | TGATATTTGA | 7 | N/A |
| 2240* | GATATTTGAT | 46 | 89 |
| 2241* | ATATTTGATT | 19 | 36 |
| 2243* | ATTTGATTAC | 12 | 13 |
| 2247* | GATTACCAAG | 14 | 12 |
| 2251* | ACCAAGTCTG | 98 | 120 |
| 2253 | CAAGTCTGTG | N/A | 9 |
| 2254* | AAGTCTGTGC | 216 | 457 |
| 2268 | TGATTGAATG | N/A | 10 |
| 2269 | GATTGAATGT | N/A | 20 |
| 2272 | TGAATGTCAG | 11 | N/A |
| 2273* | GAATGTCAGC | 19 | 35 |
| 2274* | AATGTCAGCT | 71 | 134 |
| 2280* | AGCTCCATAG | 7 | 16 |
| 2309* | ATTGGAGGTC | 7 | 12 |
| 2373 | AGATCATTGT | N/A | 8 |
| 2411* | ACTTGGATTT | 81 | 82 |
| 2416* | GATTTGTGGG | 9 | 10 |
| 2417* | ATTTGTGGGC | 10 | 15 |
| 2420 | TGTGGGCTTG | N/A | 6 |
| 2433 | TATGCTGGGC | N/A | 26 |
| 2434* | ATGCTGGGCT | 60 | 103 |
| 2464* | AAAAAAAAGA | 14 | 24 |
| 2472 | GATGCTAAAA | N/A | 8 |
| 2499 | ATAGATTTGA | 15 | 15 |
|  |  |  |  |
| *PtBTF3.2*  3′-UTR Position：2786 | | | |
| Position | 10-nt window | Experiment1  Reads | Experiment2  Reads |
| 5′-UTR/intron/exons | | | |
| 2232* | ACAAACTAAG | 10 | 16 |
| 2234* | AAACTAAGAG | 36 | 255 |
| 2239 | AAGAGTATGG | 7 | N/A |
| 2242 | AGTATGGCTC | 7 | N/A |
| 2415 | AACTCATTTA | N/A | 236 |
| 2630* | AAGAAATTGG | 61 | 38 |
| 2633 | AAATTGGCGG | 6 | N/A |
| 3′-UTR | | | |
| 2875 | TTATGTTCTC | N/A | 8 |
| 2876* | TATGTTCTCT | 24 | 54 |
| 2877* | ATGTTCTCTG | 89 | 124 |
| 2882 | CTCTGTGACT | 9 | N/A |
| 2883 | TCTGTGACTC | 18 | 34 |
| 2885 | TGTGACTCAT | N/A | 8 |
| 2889 | ACTCATAATG | N/A | 23 |
| 2891 | TCATAATGGT | N/A | 25 |
| 2892 | CATAATGGTT | N/A | 20 |
| 2893* | ATAATGGTTG | 76 | 166 |
| 2895* | AATGGTTGTT | 16 | 16 |
| 2911 | AGTTTTCGAT | N/A | 14 |
| 2923* | AATATACTTT | 50 | 151 |
| 2926* | ATACTTTTTC | 21 | 32 |
| 2935* | CTGAAATTCG | 307 | 555 |
| 2936 | TGAAATTCGT | 13 | N/A |
| 2937* | GAAATTCGTG | 17 | 26 |
| 2938* | AAATTCGTGA | 223 | 308 |
| 2943 | CGTGATTGAT | 9 | N/A |
| 2946 | GATTGATATT | N/A | 7 |
| 2947 | ATTGATATTT | N/A | 9 |
| 2948 | TTGATATTTT | 7 | N/A |
| 2949* | TGATATTTTC | 28 | 38 |
| 2950* | GATATTTTCC | 218 | 260 |
| 2951* | ATATTTTCCT | 99 | 238 |
| 2953* | ATTTTCCTGG | 134 | 154 |
| 2965 | GAATGTCAGC | N/A | 9 |
| 2966* | AATGTCAGCA | 17 | 28 |
| 3011* | AAAAAAAATT | 50 | 47 |
| 3048 | GAAAAGCTCT | N/A | 12 |
| 3049 | AAAAGCTCTT | N/A | 20 |
|  |  |  |  |
| *PtNAC002*  3′-UTR Position：1285 | | | |
| Position | 10-nt window | Experiment1  Reads | Experiment2  Reads |
| 5′-UTR/intron/exons | | | |
| 742 | AAGAAAAACA | N/A | 793 |
| 3`UTR | | | |
| 1380 | ACAAGAGAAA | 57 | N/A |
| 1382 | AAGAGAAAGT | N/A | 106 |
| 1434 | AAATGGACTC | 170 | N/A |
| 1561 | ATCATCAACA | N/A | 106 |
| 1634* | AAAATCCCTT | 189 | 88 |
| 1691 | GGCTATCGTA | 312 | N/A |
| 1743 | AAAAAAAGAA | 158 | N/A |
|  |  |  |  |
| *PtNAC015*  3′-UTR Position：1748 | | | |
| Position | 10-nt window | Experiment1  Reads | Experiment2  Reads |
| 5′-UTR/intron/exons | | | |
| 384* | AGATAAAGAA | 554 | 98 |
| 385* | GATAAAGAAA | 175 | 51 |
| 386* | ATAAAGAAAA | 7270 | 1485 |
| 387* | TAAAGAAAAA | 237 | 58 |
| 388* | AAAGAAAAAA | 50808 | 13072 |
| 389* | AAGAAAAAAA | 182 | 50 |
| 390* | AGAAAAAAAT | 62 | 15 |
| 391* | GAAAAAAATA | 160 | 63 |
| 392* | AAAAAAATAG | 17447 | 1924 |
| 428 | AAAACAAGAC | 520 | N/A |
| 541 | GAAGAGCTTA | N/A | 10 |
| 542 | AAGAGCTTAT | N/A | 1401 |
| 869 | AAAGATAAGG | 297 | N/A |
| 892 | AAAATCCCTG | 171 | N/A |
| 906* | GAATGAAAAA | 191 | 12 |
| 907 | AATGAAAAAG | 195 | N/A |
| 911 | AAAAAGACCC | 75 | N/A |
| 946* | TAAAGGAGAG | 72 | 30 |
| 947* | AAAGGAGAGA | 475 | 636 |
| 952 | AGAGAAAACC | N/A | 45 |
| 1019 | AAAACTGCCA | 299 | N/A |
| 1178 | GGAAAGAAGA | 249 | N/A |
| 1180 | AAAGAAGACC | N/A | 11 |
| 1338 | ATGATCAAAG | 59 | N/A |
| 1341 | ATCAAAGAAA | 188 | N/A |
| 1666 | AAAAAGGTCA | N/A | 12 |
| 3`UTR | | | |
| 1788 | AAGAAAAAGA | N/A | 13 |
| 1791* | AAAAAGAAGA | 113 | 195 |
| 1873 | ATAAATTGAA | 10 | N/A |
| 1875* | AAATTGAAAG | 8 | 46 |
| 1887* | AGTGAACAAT | 13 | 28 |
| 1891 | AACAATGTTG | N/A | 13 |
| 1902 | ATGTATTGTA | N/A | 18 |
| 1906 | ATTGTAAGAA | 17 | N/A |
| 1911* | AAGAAATTAT | 63 | 65 |
| 1914* | AAATTATCTT | 21 | 9 |
| 1924* | ATGAATTATC | 35 | 15 |
| 1927 | AATTATCTCT | N/A | 8 |
| 1931* | ATCTCTTCCT | 155 | 96 |
| 1936* | TTCCTCCTCC | 103 | 231 |
| 1941* | CCTCCTCGTC | 16 | 13 |
| 1942* | CTCCTCGTCG | 14 | 53 |
| 1943* | TCCTCGTCGT | 264 | 432 |
| 1944* | CCTCGTCGTT | 211 | 399 |
| 1945* | CTCGTCGTTG | 83 | 175 |
| 1946* | TCGTCGTTGT | 72 | 142 |
| 1947* | CGTCGTTGTC | 185 | 174 |
| 1948 | GTCGTTGTCT | 21 | N/A |
| 1951* | GTTGTCTATA | 11 | 36 |
| 1954 | GTCTATATCT | N/A | 9 |
| 1957* | TATATCTGTT | 17 | 26 |
| 1958* | ATATCTGTTG | 138 | 146 |
| 1960* | ATCTGTTGGG | 14 | 23 |
| 2009* | AGAATCTGTC | 14 | 17 |
| 2084* | AATAAGTGCT | 34 | 96 |
| 2087* | AAGTGCTTAG | 69 | 44 |
| 2101 | TCCATTATTG | 21 | N/A |
| 2104* | ATTATTGAGA | 17 | 139 |
| 2107* | ATTGAGATTA | 23 | 8 |
| 2111* | AGCATAATCT | 36 | 36 |
| 2170 | AATTTGGGAT | N/A | 13 |
|  |  |  |  |
| *PtNAC028*  3′-UTR Position：1496 | | | |
| Position | 10-nt window | Experiment1  Reads | Experiment2  Reads |
| 5′-UTR/intron/exons | | | |
| 229* | GAAAACAATT | 672 | 46 |
| 230* | AAAACAATTG | 2019 | 1077 |
| 235 | AATTGTGTGT | 2729 | N/A |
| 529 | TGAAAAGGAA | 76 | N/A |
| 530* | GAAAAGGAAT | 137 | 66 |
| 531* | AAAAGGAATG | 166 | 492 |
| 629* | AAAGACAAGG | 60 | 579 |
| 639 | AAATTTACAG | 28 | N/A |
| 707* | AAAGGAGAAA | 2597 | 6208 |
| 712* | AGAAAAAACC | 829 | 179 |
| 714* | AAAAAACCAA | 246 | 1117 |
| 778 | TAAAACAGCA | N/A | 11 |
| 779 | AAAACAGCAA | N/A | 20 |
| 787* | AAAGGTAGGG | 164 | 216 |
| 942* | GAGGAAAGAA | 47 | 14 |
| 943* | AGGAAAGAAA | 77 | 289 |
| 944 | GGAAAGAAAA | 54 | N/A |
| 946 | AAAGAAAACA | N/A | 120 |
| 990* | GAAATGAATT | 100 | 103 |
| 991* | AAATGAATTA | 85 | 868 |
| 3′-UTR | | | |
| 1517* | TTAAAAAAAA | 126 | 126 |
| 1518* | TAAAAAAAAA | 316 | 203 |
| 1519* | AAAAAAAAAA | 2256 | 3391 |
| 1712* | ACGCAAATTT | 26 | 17 |
| 1716* | AAATTTGTTA | 9 | 22 |
| 1725 | AACATTAGGA | 9 | N/A |
| 1773* | AGTTCTTGAG | 53 | 62 |
| 1809* | AAGAGTCTGA | 37 | 28 |
| 1892* | AAAAAAAAAA | 30 | 40 |
|  |  |  |  |
| *PtNAC035*  3′-UTR Position：3263 | | | |
| Position | 10-nt window | Experiment1  Reads | Experiment2  Reads |
| 5′-UTR/intron/exons |  |  |  |
| 219 | TGGCCGCCTG | 61 | N/A |
| 221* | GCCGCCTGGG | 321 | 32 |
| 224* | GCCTGGGTTC | 18 | 9 |
| 274 | TGAAGAAGAA | 143 | N/A |
| 275 | GAAGAAGAAG | 285 | N/A |
| 276 | AAGAAGAAGA | 1655 | N/A |
| 279 | AAGAAGATCT | 119 | N/A |
| 291 | AAGAAAAGGA | 1190 | N/A |
| 294 | AAAAGGATAA | 5542 | N/A |
| 1232 | GATGAAGAAG | 416 | N/A |
| 1233 | ATGAAGAAGA | 1471 | N/A |
| 1388 | AAAAAAAGTG | 87 | N/A |
| 1599 | AAATAGCTGA | 31 | N/A |
| 3′-UTR | | | |
| 3331 | ATAAAAATGG | N/A | 83 |
| 3333 | AAAAATGGTT | N/A | 385 |
| 3352 | AATTATAGTA | N/A | 27 |
| 3356 | ATAGTAGTTT | N/A | 37 |
| 3358 | AGTAGTTTCG | N/A | 26 |
| 3360 | TAGTTTCGGT | N/A | 18 |
| 3361 | AGTTTCGGTT | N/A | 174 |
| 3377 | ATATCATCTT | N/A | 24 |
| 3418 | TGAACTGTTG | N/A | 28 |
| 3419 | GAACTGTTGA | N/A | 12 |
| 3420 | AACTGTTGAA | N/A | 37 |
| 3526 | ATGCTCATTG | N/A | 17 |
|  |  |  |  |
| *PtNAC052*  3′-UTR Position：1686 | | | |
| Position | 10-nt window | Experiment1  Reads | Experiment2  Reads |
| 5′-UTR/intron/exons | | | |
| 342 | TGGGTTGAGA | 5 | N/A |
| 387 | AAAGTTTTTG | N/A | 4 |
| 400 | AATTTCACGT | N/A | 30 |
| 455 | AAGAATGGTG | 146 | N/A |
| 484* | ATTTAGGTTC | 7 | 4 |
| 530 | AAGAGAAAGG | N/A | 4 |
| 535 | AAAGGTGTTT | N/A | 9 |
| 702* | AAAATTATCA | 37 | 40 |
| 723 | ATTTTTAGAG | 76 | N/A |
| 728 | TAGAGATAGA | N/A | 18 |
| 731 | AGATAGATTG | 118 | N/A |
| 743 | AATTAAAACC | 15 | N/A |
| 752 | CAAAATGCAT | 24 | N/A |
| 753* | AAAATGCATT | 377 | 47 |
| 757 | TGCATTGCCA | 8 | N/A |
| 760* | ATTGCCACGT | 15 | 45 |
| 777 | ACACCACTTG | N/A | 6 |
| 779 | ACCACTTGAG | 6 | N/A |
| 801* | AAGAAAATTC | 4 | 5 |
| 804 | AAAATTCAAG | 5 | N/A |
| 815 | AGATAATTGC | N/A | 117 |
| 865 | TTGGAGCAGG | 26 | N/A |
| 869 | AGCAGGAGAG | N/A | 18 |
| 870 | GCAGGAGAGG | N/A | 78 |
| 875 | AGAGGTACTT | 40 | N/A |
| 895 | AGGGAAGCCA | 4 | N/A |
| 897 | GGAAGCCAAG | 74 | N/A |
| 966* | AATAGACAAG | 9 | 8 |
| 1011 | GATGAAGAAA | 4 | N/A |
| 1012 | ATGAAGAAAA | N/A | 127 |
| 1120* | GCCCTGCTAA | 13 | 19 |
| 1125* | GCTAAATAAG | 7 | 12 |
| 1126* | CTAAATAAGA | 24 | 57 |
| 1227 | CACTGAGATT | 8 | N/A |
| 1228* | ACTGAGATTC | 8 | 5 |
| 1230 | TGAGATTCAT | 5 | N/A |
| 1231* | GAGATTCATC | 30 | 7 |
| 1232* | AGATTCATCT | 87 | 68 |
| 1234 | ATTCATCTCT | 5 | N/A |
| 1238 | ATCTCTCTGT | 9 | N/A |
| 1275 | TAAAAAAAAA | 15 | N/A |
| 1276* | AAAAAAAAAA | 423 | 135 |
| 1403* | AATGGAAAAT | 4 | 9 |
| 1407* | GAAAATTGGG | 10 | 6 |
| 1408* | AAAATTGGGT | 139 | 54 |
| 1437* | AAGAAGAGGA | 14 | 7 |
| 1452* | AAAAATGAGG | 36 | 29 |
| 1459 | AGGAAGAAAA | 7 | N/A |
| 1462 | AAGAAAACAT | 17 | N/A |
| 1465 | AAAACATGCA | 9 | N/A |
| 3′-UTR | | | |
| 1706* | AGCCATTAGT | 11 | 26 |
| 1710* | ATTAGTTCTT | 6 | 12 |
| 1725* | GTATTTAGGT | 7 | 8 |
| 1726* | TATTTAGGTG | 14 | 13 |
| 1727* | ATTTAGGTGT | 83 | 38 |
| 1769* | CAAACTAATG | 10 | 5 |
| 1770* | AAACTAATGC | 259 | 262 |
| 1774 | TAATGCTTGT | 14 | N/A |
| 1775* | AATGCTTGTG | 272 | 159 |
| 1785 | TCCTTAGGTT | 10 | N/A |
| 1786 | CCTTAGGTTT | N/A | 6 |
| 1787* | CTTAGGTTTC | 58 | 64 |
| 1788* | TTAGGTTTCG | 19 | 33 |
| 1789* | TAGGTTTCGT | 44 | 39 |
| 1790* | AGGTTTCGTC | 169 | 107 |
| 1795 | TCGTCTCTCT | N/A | 4 |
| 1796* | CGTCTCTCTC | 8 | 8 |
| 1800 | TCTCTCATCT | 8 | N/A |
| 1802 | TCTCATCTGT | 5 | N/A |
| 1840* | ACAAGAAAAA | 14 | 5 |
| 1842* | AAGAAAAAAA | 302 | 366 |
| 1843 | AGAAAAAAAA | 9 | N/A |
| 1844 | GAAAAAAAAA | N/A | 6 |
| 1845* | AAAAAAAAAA | 1203 | 843 |
| 1849* | AAAAAAGGAA | 8 | 8 |
| 1854* | AGGAAAAGAA | 11 | 17 |
| 1857* | AAAAGAAAAC | 38 | 12 |
| 1954* | AATTGATATT | 97 | 16 |
| 1964 | TCTCTCTTTT | 11 | N/A |
| 1966 | TCTCTTTTTG | 15 | N/A |
| 1970* | TTTTTGCAAA | 6 | 6 |
| 1975* | GCAAATAGAA | 29 | 5 |
| 1976* | CAAATAGAAA | 40 | 15 |
| 1977* | AAATAGAAAT | 1303 | 522 |
| 1979 | ATAGAAATGT | N/A | 6 |
| 1980 | TAGAAATGTT | N/A | 4 |
| 1981* | AGAAATGTTT | 160 | 33 |
| 1983* | AAATGTTTCT | 29 | 7 |
| 1995 | AATCATGTTT | 9 | N/A |
| 1999* | ATGTTTAAGC | 30 | 6 |
|  |  |  |  |
| *PtNAC053*  3′-UTR Position：1798 | | | |
| Position | 10-nt window | Experiment1  Reads | Experiment2  Reads |
| 5′-UTR/intron/exons | | | |
| 196 | CCTCAAACAC | 587 | N/A |
| 545 | TCATAAGGGT | 13 | N/A |
| 546 | CATAAGGGTA | 23 | N/A |
| 547 | ATAAGGGTAT | 19 | N/A |
| 548 | TAAGGGTATG | 7 | N/A |
| 651* | AAGAGAAAGG | 26 | 15 |
| 654 | AGAAAGGTGT | N/A | 13 |
| 656 | AAAGGTGTTT | N/A | 317 |
| 676 | TGCCTGCTTC | 39 | N/A |
| 805 | ATCTTTGTAG | N/A | 7 |
| 844 | AAAGGCATTT | 13 | N/A |
| 856 | AAAGATAGAT | 72 | N/A |
| 874* | AAAACCAAAC | 46 | 165 |
| 880 | AAACTGCAAT | 12 | N/A |
| 890 | GTCATGTGGC | 27 | N/A |
| 915 | AAACGAAGTT | 39 | N/A |
| 920 | AAGTTGCAAG | 11 | N/A |
| 925 | GCAAGAAAAA | N/A | 18 |
| 927* | AAGAAAAATT | 282 | 264 |
| 930* | AAAAATTACG | 95 | 32 |
| 942 | AAGATAATTT | 26 | N/A |
| 945 | AATTTCTACT | 25 | N/A |
| 965 | ATATGGCGTT | 22 | N/A |
| 998* | AAGAACGGTA | 20 | 37 |
| 1023 | AGAAGCCAAA | 19 | N/A |
| 1092 | AATAGACAAG | N/A | 7 |
| 1247 | CCCTGAAAAA | N/A | 8 |
| 1250 | TGAAAAATAA | N/A | 110 |
| 1251* | GAAAAATAAA | 164 | 157 |
| 1252* | AAAAATAAAA | 1064 | 1778 |
| 1258* | AAAAATTCAA | 60 | 177 |
| 1384* | TGAAAAAGAA | 803 | 699 |
| 1385* | GAAAAAGAAA | 618 | 390 |
| 1386* | AAAAAGAAAA | 1612 | 2431 |
| 1392 | AAAAAACCTT | N/A | 6 |
| 1647* | AAAGGAGAAG | 11 | 6 |
| 1783 | AGAAGAAAAC | N/A | 13 |
| 1785 | AAGAAAACCA | 18 | N/A |
| 3′-UTR | | | |
| 1883* | AATCTAATGC | 22 | 11 |
| 1888* | AATGCTTGTG | 38 | 33 |
| 1900* | TTTCGGTTTC | 65 | 96 |
| 1904 | CGGTTTCGTC | 11 | N/A |
| 1908* | TTCGTCTCTC | 12 | 85 |
| 1909* | CTTCTCTCTG | 168 | 184 |
| 1910* | GTCTCTCTCA | 22 | 27 |
| 1911* | TCTCTCTCAT | 18 | 22 |
| 1913* | TCTCTCATCT | 93 | 130 |
| 1914* | CTCTCATCTG | 10 | 12 |
| 1915* | TCTCATCTGT | 32 | 52 |
| 1918 | CATCTGTGAGTG | N/A | 13 |
| 1919* | ATCTGTGAGT | 8 | 14 |
| 1957 | AAGAAAAGAA | N/A | 6 |
| 1960* | AAAAGAAAAG | 11 | 14 |
| 1965* | AAAAGAAAAA | 160 | 179 |
| 1970* | AAAAACAAAC | 37 | 34 |
| 2086* | TAGTGATATT | 20 | 17 |
| 2087* | AGTGATATTT | 112 | 117 |
| 2093* | ATTTCTCCTC | 27 | 6 |
| 2101* | TCCTTTGCAA | 19 | 36 |
| 2103* | CTTTGCAAAT | 74 | 96 |
| 2104 | TTTGCAAATGGA | N/A | 19 |
| 2108 | CAAATGGAAAT | N/A | 51 |
| 2109* | AAATGGAAAT | 1702 | 2145 |
| 2114* | GAAATGTTTC | 30 | 30 |
| 2115* | AAATGTTTCA | 318 | 240 |
| 2127* | AAACATGTTT | 28 | 22 |
| 2131* | ATGTTTAAGC | 20 | 9 |
| 2144* | AATTTTACGC | 12 | 8 |
|  |  |  |  |
| *PtNAC061*  3′-UTR Position：1489 | | | |
| Position | 10-nt window | Experiment1  Reads | Experiment2  Reads |
| 5′-UTR/intron/exons | | | |
| 322 | TAAAAGAAAT | 61 | N/A |
| 323* | AAAAGAAATC | 923 | 29 |
| 452* | CAAAATAGGA | 77 | 95 |
| 453* | AAAATAGGAA | 3103 | 5605 |
| 472* | AAAACGATTG | 112 | 25 |
| 602 | GGTTGGAATG | N/A | 26 |
| 603 | GTTGGAATGA | N/A | 42 |
| 855* | CAAGAAGAAA | 26 | 95 |
| 856* | AAGAAGAAAA | 940 | 2196 |
| 859* | AAGAAAAATC | 81 | 89 |
| 862* | AAAAATCTCA | 564 | 1280 |
| 994* | AAAGAAGAAA | 394 | 3266 |
| 1441 | AGATCATCGT | N/A | 40 |
| 3′-UTR | | | |
| 1524* | AAAAGACAGA | 80 | 82 |
| 1603 | ATAAGAGTTC | N/A | 19 |
| 1612 | CTTAGATGGG | 13 | N/A |
| 1650 | TATCAAGATG | N/A | 44 |
| 1651* | ATCAAGATGT | 27 | 143 |
| 1673* | AATTTGTTTT | 38 | 24 |
| 1733 | AACTGCGCAT | 26 | N/A |
| 1761 | ATTCTTCTCT | 53 | N/A |
| 1764 | CTTCTCTTGC | 30 | N/A |
| 1765* | TTCTCTTGCC | 47 | 16 |
| 1766* | TCTCTTGCCT | 43 | 37 |
|  |  |  |  |
| *PtNAC065*  3′-UTR Position：1595 | | | |
| Position | 10-nt window | Experiment1  Reads | Experiment2  Reads |
| 5′-UTR/intron/exons | | | |
| 478 | AAGTGATGTT | 14 | N/A |
| 523* | AAAATAGGAA | 238 | 186 |
| 542* | AAAATGATTG | 51 | 8 |
| 893 | GTCTAGGTCT | 9 | N/A |
| 960* | TCAAGAAGAA | 312 | 1123 |
| 961 | CAAGAAGAAA | 341 | N/A |
| 962 | AAGAAGAAAA | 3833 | N/A |
| 964 | GAAGAAAAAC | 15 | N/A |
| 965 | AAGAAAAACC | 324 | N/A |
| 968 | AAAAACCTCA | 669 | N/A |
| 1099 | CAAAGAAGAA | 23 | N/A |
| 1100 | AAAGAAGAAA | 957 | N/A |
| 3′-UTR | | | |
| 1739* | AGATGCGATG | 38 | 50 |
| 1741* | ATGCGATGAC | 35 | 44 |
| 1745* | GATGACACTG | 86 | 103 |
| 1746* | ATGACACTGT | 23 | 31 |
| 1774* | AATCAAGATG | 16 | 27 |
| 1797* | AATTTGTTTT | 80 | 79 |
|  |  |  |  |
| *PtNAC074*  3′-UTR Position：1577 | | | |
| Position | 10-nt window | Experiment1  Reads | Experiment2  Reads |
| 5′-UTR/intron/exons | | | |
| 382 | GTCAAAGGTT | N/A | 49 |
| 383 | TCAAAGGTTG | N/A | 6849 |
| 385 | AAAGGTTGGG | 115 | N/A |
| 387 | AGGTTGGGAG | 1397 | N/A |
| 440 | AACGGAGGAA | 12 | N/A |
| 797 | AAAAGAAAAG | 138 | N/A |
| 809 | AAAAGGAAAT | 47 | N/A |
| 832 | AAAAAAACAC | 24 | N/A |
| 1248 | AAGAAGAAAT | 208 | N/A |
| 1251 | AAGAAATGAA | 94 | N/A |
| 3′-UTR | | | |
| 1609* | AGATAACTTC | 29 | 95 |
| 1630 | AATTATCAAC | N/A | 127 |
| 1748 | AATGAACTAC | N/A | 37 |
| 1764 | AAGAATTTAA | N/A | 21 |
| 1767* | AATTTAATTT | 17 | 21 |
| 1771* | TAATTTACCA | 12 | 8 |
| 1772* | AATTTACCAT | 344 | 610 |
| 1776* | TACCATTGCT | 21 | 58 |
| 1777* | ACCATTGCTC | 388 | 780 |
| 1780* | ATTGCTCTGA | 95 | 236 |
| 1783* | GCTCTGAATA | 78 | 25 |
| 1787* | TGAATAATCA | 60 | 102 |
| 1788* | GAATAATCAA | 99 | 76 |
| 1789* | AATAATCAAT | 622 | 1704 |
| 1792* | AATCAATCCT | 144 | 242 |
| 1796* | AATCCTCAAA | 28 | 73 |
| 1803* | AAATACTCCC | 18 | 47 |
| 1818 | TGAAGTGGAA | 12 | N/A |
| 1819 | GAAGTGGAAT | N/A | 37 |
| 1820* | AAGTGGAATG | 79 | 82 |
| 1826 | AATGTCGTTG | N/A | 110 |
| 1840 | ATGTCGATAC | 15 | N/A |
| 3057* | AAAGTCGTGG | 12 | 15 |
|  |  |  |  |
| *PtNAC113*  3′-UTR Position：1380 | | | |
| Position | 10-nt window | Experiment1  Reads | Experiment2  Reads |
| 5′-UTR/intron/exons | | | |
| 338 | AAACAAAAGG | 11875 | N/A |
| 815* | ATTGAAGAAA | 462 | 10 |
| 844 | GAAAAGCTCC | N/A | 14 |
| 845* | AAAAGCTCCG | 806 | 1582 |
| 859 | GAAGAAAAAC | 53 | N/A |
| 860 | AAGAAAAACC | 2822 | N/A |
| 863* | AAAAACCAAA | 50 | 11 |
| 3′-UTR | | | |
| 1433 | AAATTCTTTT | 26 | N/A |
| 1449 | ATTTAATAGG | 89 | N/A |
| 1453* | AATAGGAGGA | 188 | 36 |
| 1459 | AGGAAAGTTA | 41 | N/A |
| 1474* | AATGAAAAAA | 35 | 69 |
| 1476 | TGAAAAAAAA | 19 | N/A |
| 1478* | AAAAAAAAAA | 149 | 242 |
| 1604 | AGGAGAAAAT | 22 | N/A |
| 1627 | TCTAACCAGT | 41 | N/A |
| 1629 | TAACCAGTTT | N/A | 21 |
| 1647 | AACCTAGAAT | 11 | N/A |
| 1652 | AGAATGATAG | 48 | N/A |
| 1680 | AGTTTGAAGA | 15 | N/A |
| 1887 | AAAAGAAATA | 15 | N/A |
|  |  |  |  |
| *PtATAF1.1*  3′-UTR Position：1587 | | | |
| Position | 10-nt window | Experiment1  Reads | Experiment2  Reads |
| 5′-UTR/intron/exons | | | |
| 227* | AAGAACAAAA | 42 | 65 |
| 230* | AACAAAAAAA | 2967 | 2963 |
| 231 | ACAAAAAAAT | 10 | N/A |
| 232* | CAAAAAAATG | 133 | 27 |
| 233* | AAAAAAATGA | 10350 | 13135 |
| 234* | AAAAAATGAC | 12 | 10 |
| 235* | AAAAATGACA | 60 | 61 |
| 236 | AAAATGACAG | 25 | N/A |
| 237 | AAATGACAGC | 29 | N/A |
| 238 | AATGACAGCG | 12 | N/A |
| 239 | ATGACAGCGG | 12 | N/A |
| 486 | AAATTACAGG | 11 | N/A |
| 510* | GAGAAAAGGA | 28 | 14 |
| 511* | AGAAAAGGAA | 584 | 156 |
| 513* | AAAAGGAATG | 103 | 28 |
| 673 | AGGAAAAGCT | 18 | N/A |
| 675 | GAAAAGCTCC | 166 | N/A |
| 676 | AAAAGCTCCC | 1085 | N/A |
| 686 | AAGGGAGAGA | 10 | N/A |
| 690 | GAGAGAAAAC | 76 | N/A |
| 691 | AGAGAAAACG | 422 | N/A |
| 693 | AGAAAACGAA | 92 | N/A |
| 695 | AAAACGAACT | 18 | N/A |
| 752 | AAGAAGAACA | 43 | N/A |
| 861 | AGCTTTGGCC | 114 | N/A |
| 879 | ACGATACTTG | 107 | N/A |
| 881 | GATACTTGGA | 17 | N/A |
| 882 | ATACTTGGAT | 36 | N/A |
| 917 | ACATATTTTT | 22 | N/A |
| 928* | GCAAAAATTG | 16 | 14 |
| 929* | CAAAAATTGT | 81 | 41 |
| 930* | AAAAATTGTG | 3401 | 2191 |
| 945* | AAATTTGGTG | 80 | 15 |
| 961 | AGATGCTGCT | N/A | 11 |
| 1104 | ATAAGACTGG | 29 | N/A |
| 1124 | ATTTTGTGTG | 28 | N/A |
| 1213* | ACAACAAGAA | 94 | 243 |
| 1215* | AACAAGAAAG | 608 | 8974 |
| 1218* | AAGAAAGGCA | 296 | 451 |
| 1227 | ACAATTGAGA | 56 | N/A |
| 1229 | AATTGAGAAG | 422 | N/A |
| 3′-UTR | | | |
| 1882 | ATTAATCTGC | 25 | N/A |
| 1885* | AATCTGCACG | 371 | 116 |
| 1889 | TGCACGTATA | 40 | N/A |
| 1892* | ACGTATATCA | 107 | 31 |
| 1895* | TATATCATTT | 97 | 20 |
| 1896* | ATATCATTTG | 860 | 326 |
| 1909* | AATCTGTGTT | 206 | 186 |
| 1953 | TGTTCTTGAA | 10 | N/A |
|  |  |  |  |
| *PtATAF1.2*  3′-UTR Position：1539 | | | |
| Position | 10-nt window | Experiment1  Reads | Experiment2  Reads |
| 5′-UTR/intron/exons | | | |
| 335* | AATTTCTCAA | 34 | 69 |
| 342 | CAAAAAACTA | N/A | 8 |
| 343* | AAAAAACTAG | 11138 | 1203 |
| 372 | TTAACTGTTT | 10 | N/A |
| 411* | AGAAAGGAAT | 42 | 42 |
| 413 | AAAAGGAATG | N/A | 18 |
| 439 | AGAGACAGGA | 64 | N/A |
| 445 | AGGAAGTACC | 19 | N/A |
| 546* | AATCAAGAAA | 19 | 76 |
| 573* | GGGGAAAGCT | 161 | 149 |
| 575 | GGAAAGCTCC | 6 | N/A |
| 576 | GAAAGCTCCT | N/A | 20 |
| 577* | AAAGCTCCTA | 45 | 94 |
| 591* | AGAGAAAACG | 213 | 778 |
| 593* | AGAAAACGAA | 41 | 12 |
| 595* | AAAACGAACT | 30 | 40 |
| 652* | AAGAAGAACA | 37 | 204 |
| 754 | GAAATGACTT | 7 | N/A |
| 755* | AAATGACTTT | 12 | 12 |
| 760* | ACTTTTTTGG | 39 | 14 |
| 762 | TTTTTTGGCC | 9 | N/A |
| 777 | ATTACGATAG | 7 | N/A |
| 780* | ACGATAGTTG | 9 | 8 |
| 800* | AATTATTAAC | 32 | 14 |
| 811 | TAATCACATA | N/A | 7 |
| 812* | AATCACATAC | 94 | 43 |
| 828* | AATGATGCTG | 134 | 42 |
| 832 | ATGCTGATTA | 11 | N/A |
| 841 | AATCCCTAAA | 7 | N/A |
| 848 | AAAATCAAGT | 7 | N/A |
| 913* | TGAAGTTAAA | 8 | 15 |
| 914* | GAAGTTAAAA | 132 | 387 |
| 915* | AAGTTAAAAA | 404 | 923 |
| 919 | TAAAAAAAGA | N/A | 58 |
| 920* | AAAAAAACAA | 220 | 230 |
| 1003 | TCCAGTTAAG | N/A | 11 |
| 1026* | ATCTCTGGAA | 28 | 42 |
| 1034 | AAAATACTTC | 23 | N/A |
| 1206* | GATGAGAAGA | 5 | 15 |
| 1207* | ATGAGAAGAA | 22 | 57 |
| 1212 | AAGAAGCAGG | 12 | N/A |
| 1392* | AAGGAATGGG | 47 | 342 |
| 3′-UTR | | | |
| 1783* | AATAGCAGAC | 97 | 82 |
| 1786* | AGCAGACTTG | 59 | 45 |
| 1789* | AGACTTGCCT | 47 | 38 |
| 1805* | AAACTTATTG | 89 | 99 |
| 1850* | AATTTGTGTT | 25 | 53 |
| 1859* | TTTTTTTTTT | 25 | 62 |
| 1860* | TTTTTTTTTT | 299 | 401 |
| 1861* | TTTTTTTTTT | 274 | 336 |
| 1862* | TTTTTTTTTT | 62 | 86 |
| 1863* | TTTTTTTTTT | 64 | 107 |
| 1864* | TTTTTTTTTT | 36 | 48 |
| 1865* | TTTTTTTTTT | 13 | 22 |
| 1866* | TTTTTTTTTT | 9 | 21 |
| 1869* | TTTTTTTTTT | 36 | 42 |

a, the position on gDNA sequence of target genes

*, reproducible candidate PASs

**Table S5** Primers used in experiments

| Primers used in 3′-RACE | |
| --- | --- |
| Primer | Sequence |
| PtNAC028-outer | CCTATCACAGCCTTTTCGGTTCA |
| PtNAC028-inner | CAGGCTTTCATCTACAAGAGCTTGGTTTGGC |
| PtATAF1.1-outer | AGAAATAATCCCTGCCGAGTC |
| Pt-ATAF1.1-inner | TTCAAGCGCCGTCACTGTATTAGACCCACCG |
| PtNAC002-outer | GATTCATCGTCTGGGTCGCAGCAC |
| PtNAC002-inner | AGAAGGTTGCATCAGTTCCATTACCGGTTAC |
| PtNAC053- outer | TGAGCCACAAGGCACATACATAC |
| PtNAC053- inner | GTTCCTTTTGAAGTTCTTGTGAGGTGCTTTC |
| PtNAC052- outer | CCTGTTCTTGTTGAGGCTCTTTG |
| PtNAC052- inner | TGATGGTGGGTTGTGACTATAAGAAGGGTGC |
| PtNAC061&065- outer | TGAATGGGCAATCTCAGGTCC |
| PtNAC061&065- inner | CGTATGAGAAGATTGACTTAGAAGTGATCCGAGAT |
| PtNAC074- outer | TTTTCAATTTCATATCGAGAATAGC |
| PtNAC074- inner | AGTAGTCACTAACCACTATAGACTGTCTGCC |
| PtNAC035- outer | GCGTGACCAACAAATCAACTC |
| PtNAC035- inner | ATTCTCTATCTCCTCATCGGTCAAAAATATC |
| PtNAC113- outer | AAGAGGGAAGGTGCACTGACT |
| PtNAC113- inner | CGTGGAAGTTTCCACCAATCACAGACTTTGC |
| PtBTF3.1&3.2- outer | TGAAGATGGCCAGTGCAGTTC |
| PtBTF3.1&3.2- inner | AGTGAATGCTATACCTGCAATCGAGGAAGTCAAC |
| PtNAC015- outer | CTTCCCATAGCTTTATCACCC |
| PtNAC015- inner | GTTTGGTCAGTTCCCATTTGATACAAGAGGT |
| PtATAF1.2- outer | CTATCTCTGCCGTAAATGCTCATC |
| PtATAF1.2-inner | TGCTGTGCCTATTATTGCTGAAAT |
| 3' -RACE Outer | GCGAGCACAGAATTAATACGACT |
| 3'- RACE Inner | CGCGGATCCGAATTAATACGACTCACTATAGG |
| Primers used for verification of splice sites | |
| Primer | Sequence |
| PtNAC074sj3-F | CTTCGGAGTTGCCAGGTATA |
| PtNAC074sj3-R | CGATAAGCTCACGATTTTCC |
| PtNAC065sj2-F | ATACAAGGTGCTTCATTATCT |
| PtNAC065sj2-R | GTCTTTCTCATTCCAATCCG |
| PtNAC028sj7-F | TTGGCATGAAGAAGACCTTG |
| PtNAC028sj7-R | AATCATTCTGCAACCGAAGC |
| PtNAC074sj10-F | TTATCAACTATTGCCCCACA |
| PtNAC074sj10-R | GACTGCTACAACCAAAGACC |
| PtBTF3.1sj3-F | GAAGAAGAAGGCTATCCACA |
| PtBTF3.1sj3-R | CACTGCAAAATTAAAACCAA |
| PtNAC015sj7-F | ATTGACGGATTCTTCACCCT |
| PtNAC015sj7-R | AGCGGTCTTGAATTGAAACAGA |
| PtNAC052sj3-F | TAGTGGGTTGAGACGTTCTTG |
| PtNAC052sj3-R | ATACTTGGCTTCCCTGGTGC |
| PtNAC015sj6-F | AATGGGTGATTTGCAGGGTG |
| PtNAC015sj6-R | TCAGAAGTGGGATTCGAGGA |
| PtATAF1.2sj7-F | GGTCTGTCATCTCGGCCATCC |
| PtATAF1.2sj7-R | CCTGCTTCTTCTCATCCTCCT |
| PtNAC053sj6-F | CTCAGGTAGAGGTTTTACTGG |
| PtNAC053sj6-R | AGTTGTCTTCTCCTTTGTCAT |
| PtBTF3.2sj8-nF | GAAGAAATTGGCGGAACAGA |
| PtBTF3.2sj8-nR | GAACATAAGATTAAGAACGATG |
| PtNAC074sj6-nF | ATAGGAAACATGGCAACGGAGGA |
| PtNAC074sj6-nR | GCGTAGGCAATTCTGGTGGG |
| PtNAC035sj1-nF | CCTTTACTATTACTTTGCGTGAC |
| PtNAC035sj1-nR | TATTTCCACCAGTGCTCCTT |
| PtBTF3.1sj2-nF | TGCAATCGAGGAAGTCAACA |
| PtBTF3.1sj2-nR | AAGGTCTCCCCAGGCACAAG |
| PtBTF3.1sj10-nF | AGTCTACTGGTGCAGGTGCT |
| PtBTF3.1sj10-nR | GTAATGAATCGAAACTCTAAC |
| PtATAF1.2sj4-nF | CCTTGTATGGAGAAAAGGAATG |
| PtATAF1.2sj4-nR | AGCTGTTCTTCTTGCGAGCC |
| PtATAF1.1sj5-nF | CTGGACGATTGGGTACTCTG |
| PtATAF1.1sj5-nR | GTCGTTCCCTGAATCTACCA |
| PtNAC052sj7-nF | CCCTTGCCTGCTTCCATAAT |
| PtNAC052sj7-nR | GAGTTTTCTTCATCCCCACA |
| PtNAC028sj1-nF | TTTTATGGTTTGTTTCCTAC |
| PtNAC028sj1-nR | TCCCCTCTAATCTATACTCA |
| PtNAC028sj6-nF | TTTAAGGACGAATAGGGCTAC |
| PtNAC028sj6-nR | AAGACATATCAGTGCTGAGGC |
| Primers used in real-time RT-PCR | |
| Primer | Sequence |
| PtNAC052-FP1 | TTAGGTTTCGTCTCTCTCATCTGT |
| PtNAC052-RP1 | AACTTGTCGACATCCACATTTCT |
| PtNAC052-FP2 | TCTAGCTCCTTCATCATCCTCAG |
| PtNAC052-RP2 | ACGGTACAAGAACTAATGGCTGA |
| PtNAC052-FP3 | ATTGTGACTTCTAAGGGCAATCA |
| PtNAC052-RP3 | GTGGAAACAAGCTTCAAAGCTTT |
| Elongation factor 1-F | CCGTTGCTGTGGGAGTTATCAAG |
| Elongation factor 1-R | GGCAGCAGATTTGGTCACCTTAG |

**Table S6** Clone sequencing data of 10 canonical SSs verified by RT-PCR

| >PtNAC074 SS3  CTTCGGAGTTGCCAGGTATATGTTTGTCAAAGGTTGGGGAGAGGGAGTGGTATTTTTTTGTGCCTCGAGATAGGAAACATGGCAACGGAGGAAGGCCTAATAGGACTACACAAAATGGATTCTGGAAGGCTACTGGTTCTGATAGGAAAATCGTGAGCTTATCG  >PtNAC074 SS10  TTATCAACTATTGCCCCACATAGCTCAGCAAGTAAGAATCTTCAAGATGAGTGCTTAAGAGGGTGGCAATAACTAAATTACGTTGCCAAATCACATGGTGTCAACCAAGTTCAAAAGTCCATTTCCAAATGGTCTTTGGTTGTAGCAGTC  >PtNAC065 SS2  ATACAAGGTGCTTCATTATCTAAGAGATGCAAAATAGGAACCACCCCACAAAATGATTGGTACTTCTTTAGCCACAAGGACAAGAAATATCCAACTGGTACGCGCACCAATCGGGCAACTGCTGCTGGGTTTTGGAAGGCTACTGGCCGTGACAAGGTGATATACAGCACTGGCAAGCGGATTGGAATGAGAAAGAC  >PtNAC028 SS7  TTGGCATGAAGAAGACCTTGGTTTTCTACAAAGGGAGAGCCCCCAAAGGAGAAAAAACCAACTGGGTTATGCATGAGTATAGATTAGAGGGGAAAAACCCCGTCTATAATCCCCCTAAAACAGCAAAGATCATTTTGCCTGGCTTCGGTTGCAGAATGATT  >PtBTF3.1 SS3  GAAGAAGAAGGCTATCCACAAGACTACTACTACAGATGATAAAAGGCTTCAGAGCACCCTGAAGAGAATTGGAGTGAATGCTATACCTGCAATCGAGGAAGTCAACATATTCAAGGATGACATGGTTATCCAGTTTCTAAATCCCAAAGCTGATTATTATTTTGGTTTTAATTTTGCAGTG  >PtNAC015 SS6  AATGGGTGATTTGCAGGGTGTTTCAAAAGAGCTCGGCTGGAAAGAAGACCTATATTTCAGTTTCTTCGAACCCTTCAGAGGTTTTTCCTCGAATCCCACTTCTGA  >PtNAC015 SS7  ATTGACGGATTCTTCACCCTCCAATGGCAAGATTAGGCCGGTGGCCGAGTCGGCCTACGTGCCCTGCTTCTCCAATCTCAGTGATGATCAAAGAAACCAACAAGACACAACTGACAGCTTCAACAATCATCTTTTTGCAGTTTCTTCGAACCCTTCAGAGGTTCTGTTTCAATTCAAGACCGCT  >PtNAC052 SS3  TAGTGGGTTGAGACGTTCTTGAGCCACAAGGCACATACATATATACATAAAGTTTTTGTTCAATTTCACGTTTTTGTGAGGTGCTCTAATAAGACATGGAGAAGCTTAATTTTGTTAAGAATGGTACTTTTTCAGCACCAGGGAAGCCAAGTAT  >PtATAF1.2 SS7  GGTCTGTCATCTCGGCCATCCGTAGGGATAGTTTTGATATTTAATGGTTGAAGATGCTTCCAAATATGGACGTCGAGCTGGATGATTGGGTACTCTGTCGCATATACAACAAGAAAGGTACAGTTGAGAAGCAAGAACAGCATCTTAGCGTCAAGAAAGCGAATCCGACGGAGATTGAGGAGGATGAGAAGAAGCAGG  >PtNAC053 SS6  CTCAGGTAGAGGTTTTACTGGGGCCCTGTTGTGGTGCCAATGGAAAATTGGGTTCTATGCCGCATATTTTTGAAGAAGAGAGGCACAAAAAATGAGGAGGAAAACATTCAAGTTGGCAATGATAATAGACTGCCCAAACTCAGGGCCACTGAGCCTGTTTTCTATGATTTCATGACAAAGGAGAAGACAACT |
| --- |
